# Effect of chronic cyclic heat stress and supplemented inorganic and organic zinc source levels on grow-finish pig growth performance and estimated body composition

**DOI:** 10.1093/tas/txae029

**Published:** 2024-04-02

**Authors:** Kayla M Mills, Julie A Mahoney, Alan W Duttlinger, Sarah K Elefson, John S Radcliffe, Zachary J Rambo, Brian T Richert

**Affiliations:** US Department of Agriculture, Agricultural Research Service, Beltsville Agricultural Research Center (BARC), Animal Biosciences & Biotechnology Laboratory, Beltsville, MD 20705, USA; United Animal Health, Research and Development, Sheridan, IN 46069, USA; Country View Family Farms, Middletown, PA 17057, USA; USDA-ARS Children’s Nutrition Research Center, Department of Pediatrics, Baylor College of Medicine, Houston, TX 77030, USA; Department of Animal and Food Sciences, University of Kentucky, Lexington, KY 40506, USA; Zinpro Corporation, EdenPrairie, MN 55344, USA; Department of Animal Science, Purdue University, West Lafayette, IN 47907, USA

**Keywords:** heat stress, nutrition, swine, zinc

## Abstract

Zinc (**Zn**) supplementation has proved to mitigate the effects of heat stress with varying effects evident with Zn source during acute heat events. However, the effects of Zn supplementation during long-term summer weather patterns have yet to be explored. Therefore, the objective of this study was to identify the effects of supplementation source and level of Zn to mitigate the negative effects of long-term, cyclic heat stress in finishing swine. Six hundred cross-bred pigs were housed under thermoneutral (**TN**) or cyclic heat (**HS**) conditions simulating summer heat with acute 3-d heat waves for a 70-d study. Thermoneutral conditions were 16.7 to 18.9 °C throughout the study. HS pigs were housed at the same temperature as TN from days 0 to 18, then 28 °C/24 °C for 12 h/12 h on days 18 to 21, followed by 30 °C/26.7 °C for 12 h/12 h on days 24 to 70, except during acute heat (32 to 33 °C/29 to 30 °C, 12 h/12 h) on days 21 to 24, 42 to 45, and 63 to 66. Treatments were arranged in a 2 × 6 factorial with main effects of environment (HS vs. TN) and dietary available Zn supplementation: (1) 50 mg/kg zinc oxide (**ZnO**), (2) 130 mg/kg ZnO, (3) 50 mg/kg of organic Zn (Availa Zn), (4) 50 mg/kg ZnO + 40 mg/kg organic Zn, (5) 50 mg/kg ZnO + 60 mg/kg organic Zn, and (6) 50 mg/kg ZnO + 80 mg/kg organic Zn. Pigs (5 pigs/pen) were blocked by initial body weight (72.2 kg) and randomly allotted to 1 of 12 temperature and diet treatment combinations across 10 replicates. Body weight and feed intake were determined at the beginning and end of each acute heat event. All pigs were ultrasonically scanned at the 10th rib (**TR**) to predict loin muscle area (**LMA**), backfat (**BF**), and percent lean. Data were analyzed by the MIXED procedure in SAS with pen as the experimental unit. At day 63, HS pigs were lighter (*P* < 0.05), had lower overall average daily gain (**ADG**; *P* < 0.05) and average daily feed intake (*P* < 0.05). A diet-by-environment interaction was observed for overall ADG (*P* < 0.05) with diet 5 HS pigs having a 3.9% reduction in ADG whereas diet 6 had 14.4% reduction in ADG, while under TN temperatures diet 6 had the greatest overall ADG of all treatments. Other diets were intermediate in their ADG under both HS and TN conditions. Pigs under HS had less BF at the TR (*P* < 0.05) and a smaller LMA (*P* < 0.05), and a greater calculated percent lean (*P* < 0.05). Our results indicate that a blend of supplemental Zn sources at 50/60 mg/kg may mitigate the reduction in growth performance due to HS. While not directly contrasted, the NRC requirement of 50 mg/kg Zn may be too low to optimize finishing pig growth performance under both TN and HS conditions.

## Teaser

Feeding a blend of 50 inorganic 60 organic mg/kg Zn to finishing swine prevents the loss of growth performance under summer weather pattern simulation.

## INTRODUCTION

Heat stress has a large impact on animal production across the globe and is a growing challenge for producers due to climate change. Developing nations are particularly sensitive to heat stress as they tend to be geographically closer to the equator and have less resources readily available to cool animals (Godfray et al., 2010). In the United States, the economic loss to livestock and poultry industries ranges from $1.7 to $2.4 billion annually depending on the heat abatement strategies utilized (St-Pierre et al., 2003). In addition, heat stress can have a substantial influence on the physiological states of animals (DeShazer et al., 2009). Finishing swine are particularly sensitive to high ambient temperatures and exhibit decreased feed intake and related decreased growth rate, lower market weights, and increased fat deposition (Baumgard and Rhoads Jr, 2013; Ross et al., 2015; Mayorga et al., 2019). The swine industry loses nearly 1 billion dollars per year due to heat stress with half of those losses attributed to the grow-finish period (Pollmann, 2010; Schieck, 2019).

There are a number of ways to nutritionally mitigate heat stress in swine including increasing fat content of the diet (Spencer et al., 2005) and use of feed additives such as betaine (Kidd et al., 1997; Moeckel et al., 2002), glutamine (Lima et al., 2005), and zinc (**Zn**; Alam et al., 1994; Dardenne, 2002; Zhang and Guo, 2009; Pearce et al., 2015). Pharmacological levels of zinc oxide (**ZnO**) have been shown to increase average daily gain (**ADG**), average daily feed intake (**ADFI**), and feed efficiency (**G:F**) in weanling pigs (Hill et al., 2001). This may be due to an increase in ghrelin secretion from the stomach which stimulates appetite (Yin et al., 2009). However, it is unclear whether this mechanism holds true during the finishing phase of production. Zinc also plays a role in enterocyte regeneration, intestinal barrier function, and immune function (Alam et al., 1994; Dardenne, 2002). In finishing pigs, a blend of inorganic [60 mg/kg zinc sulfate (**ZnSO**_**4**_)] and organic zinc (60 mg/kg zinc–amino acid complex) helped maintain intestinal barrier function while animals were under acute heat stress (Pearce et al., 2015).

Zinc can be available in swine diets from either plant or supplemental sources and absorption can be influenced by a number of different factors such as the presence of phytate or similarly charged ions. Because zinc is largely unavailable to monogastrics from plant sources, zinc is supplemented in swine rations from either inorganic or organic sources. The majority of inorganic zinc sources come from either ZnO or ZnSO_4_. Zinc oxide has an estimated bioavailability from 50 to 80% and provides about 72% Zn while ZnSO_4_ is 100% available when attached to a water molecule and provides about 35.5% Zn (NRC, 2012).

Organic zinc sources are attached to either an amino acid or amino acid complex and are thought to have a greater bioavailability (O’Dell, 1969; Glover et al., 2003). Common organic sources can include but are not limited to zinc–lysine, zinc–methionine, and zinc amino–acid complexes, also known as chelates. In male guinea pigs, those fed chelated zinc grew 3% more than guinea pigs supplemented with ZnSO_4_ (Shinde et al., 2006). However, within the same study, guinea pigs fed a blend of inorganic and organic zinc were more efficient than those supplemented with ZnSO_4_ or the chelated zinc alone. However, other studies in swine noted conflicting results as to whether organic zinc sources are better for growth rate and feed intake than inorganic zinc sources (Acda and Chae, 2002; Revy et al., 2004).

In studies evaluating the effects of zinc supplementation in pigs during acute heat events, chelated Zn was shown to reduce rectal temperature and maintain intestinal integrity (Pearce et al., 2015). More specifically, 120 mg/kg organic zinc blend resulted in less overall damage to intestinal morphology, increased epithelial resistance, and decreased circulating endotoxin when compared to the pigs fed inorganic Zn at the same level (Pearce et al., 2015). These results indicate that feeding a blend of zinc sources may alleviate the negative effects associated with acute heat stress. However, little research has been conducted regarding the correct level or blend of zinc which could help mitigate some of the physiological challenges that chronic heat stress induces in swine. Therefore, this study was conducted to evaluate the effects of supplemental zinc sources and levels on finishing pigs undergoing chronic cyclical heat stress.

## MATERIALS AND METHODS

### Animals

**Figure 1.**
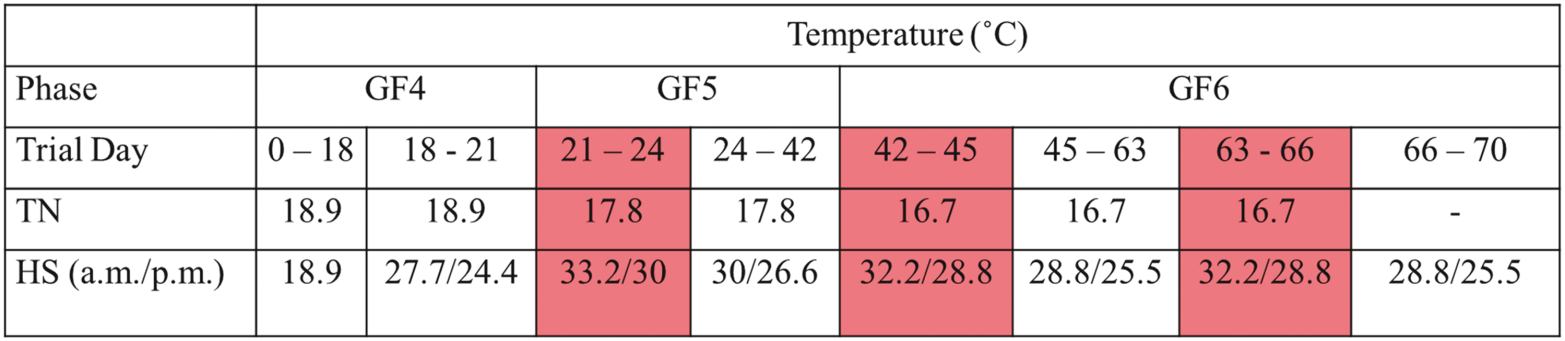
Environmental conditions and durations for TN and cyclic heat (HS) treatments. This figure outlines the temperature settings for each environment set throughout the study. Each growth phase and trial day of the study is outlined with trial Days 21-24, 42-45, and 63-66 being the elevated heat events.

**Figure 2.**
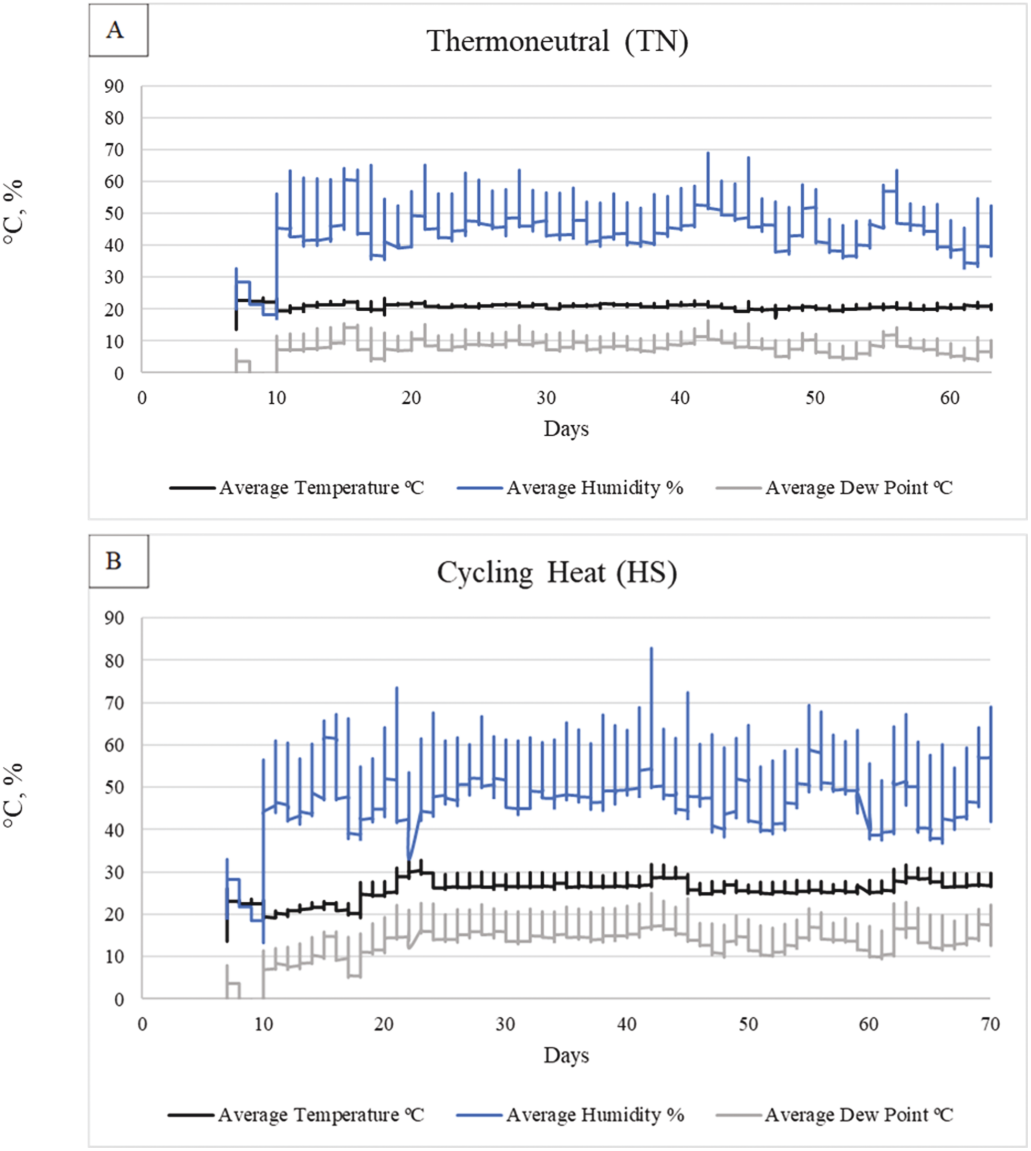
Reported average daily temperatures, humidity, and dew point. Comparison between the average daily temperature (black, middle line, °C), dew point (gray, bottom line, °C), and humidity (blue, top line, percent) of (A) TN environment and (B) cycling heat environments (HS). The horizontal axis is day of the trial and the vertical axis is °C or percent depending on the variable measured.

The experimental protocol used was approved by the Purdue University’s Institutional Animal Care and Use Committee (Protocol #1112000447). Cross-bred barrows and gilts (*n* = 600; Duroc × (York × Landrace)) were blocked by initial body weight (**BW**) of 72.2 ± 1.7 kg and sex and randomly assigned to one of six dietary treatments. Pigs were placed in 10 rooms with 5 rooms allocated to either thermoneutral (**TN**) or cyclic heat (**HS**) environments. Each room contained 12 pens and pigs within BW blocks were randomly assigned to pens (*n* = 5 mixed-sex pigs/pen) with an even sex ratio across pens in the same BW block. More specifically, the sex ratio was balanced with a mix of three barrows and two gilts within each pen, with the sex ratio flipped to two barrows and three gilts on the other side of the room. The study included three growth phases: GF4 (days 0 to 21), GF5 (days 21 to 42), and GF6 (days 42 to 70). All pigs were weighed on day 63 and TN pigs were sent to market on day 64. Pigs in the HS environment were weighed off test on day 70 and marketed on day 71 to compensate for being lighter than their TN counterparts on day 63. On day 65, one gilt per pen representative of the pen mean BW was harvested at the Purdue Animal Sciences Meat Lab for further meat quality work (Feldpausch, 2019). Presented in Figure 1 are the study design and thermal environment treatments. All pigs were fed their respective dietary treatments beginning in the acclimation period (days 0 to 18). TN conditions were 18.9, 17.8, and 16.7 °C and changed at each growth phase, respectively (Figure 2). Pigs housed under HS environment had the same temperature as TN from days 0 to 18, then increasing temperatures days 18 to 21 (28 °C/24 °C for 12 h/12 h, 50.0% humidity). On days 21 to 24 temperatures were raised to 32 to 33 °C/29 to 30 °C with humidity reaching 73.5% at its peak. Temperatures were brought back down to 29 °C/26 °C (51.5% humidity) on days 24 to 42. The second acute heat event occurred on days 42 to 45 and temperatures were returned to 32 to 33 °C/29 to 30 °C and humidity was 81%. During the third growth phase (days 45 to 63) the temperature 12 h/12 h cycle in HS rooms was 30 °C/27 °C, with 51.5% humidity except during the third and final acute heat event (days 63 to 66) when temperatures cycled to 32 to 33 °C/29 to 30 °C (66.9% humidity) and then 29 °C/25.5 °C from days 66 to 70.

### Diets

All pigs were fed corn–soybean meal-based diets with 10% DDGS and 3% added fat (Table 1). Supplemental premixes were added to basal diets to create the dietary treatments. Treatment premixes contained either inorganic Zn from ZnO, organic Zn from a Zn–amino acid complex (Availa Zinc, Zinpro Corporation, Eden Prarie, MN), or a blend of inorganic and organic Zn. The six dietary treatments were as follows: (1) 50 mg/kg available Zn from ZnO, (2) 130 mg/kg available Zn from ZnO, (3) 50 mg/kg of available Zn from organic Zn, (4) 90 mg/kg available Zn with 50 mg/kg Zn from ZnO + 40 mg/kg organic Zn, (5) 110 mg/kg available Zn with 50 mg/kg Zn from ZnO + 60 mg/kg organic Zn, and (6) 130 mg/kg available Zn with 50 mg/kg ZnO + 80 mg/kg organic Zn. Zn treatment premixes were combined with fine ground corn and calculated to be 0.20% of the complete diets, which were formulated to meet or exceed NRC (2012) nutrient requirements of growing-finishing swine (Table 1). The levels of Zn inclusion were selected to range from the minimum NRC (2012) requirement for finishing pigs (50 mg/kg) to a very high industry inclusion level (130 mg/kg) with intermediate blends of organic and inorganic Zn. All diets were made on-site at the Purdue Feedmill and were collected immediately following delivery out of bulk bins in which they were stored. Diets were then subsampled in the laboratory at Purdue University (West Lafayete, IN) and a composite by each growth phase was packaged and shipped for analysis at the University of Missouri (Columbia, MO). Diets were analyzed in triplicate at Purdue University Swine Nutrition Lab for Zn (Table 2), DM, ash, energy, N (CP), and P (Supplementary Table S1). Diets were analyzed in triplicate at the University of Missouri for amino acids, proximate analysis, Ca, P, and Zn (Supplementary Tables S2–S4). Trace mineral premixes were also analyzed by the University of Missouri Agriculture Experiment Station Chemical Laboratories (Columbia, MO) for Zn, Fe, Cu, Mn, and I. Zinc source ingredients analyzed at 77.95% Zn for ZnO and 12.49% Zn for organic Zn source.

**Table 1.** Composition of basal diets

Item	Diet Phase: GF4 (days 0 to 21)	Diet Phase: GF5 (days 21 to 42)	Diet Phase: GF6 (days 42 to 63, market)
Ingredient, %			
Corn	71.48	74.82	77.87
SBM, 48% CP	12.63	9.480	6.640
DDGS, 7% fat	10.00	10.00	10.00
Swine grease	3.000	3.000	3.000
Limestone	1.290	1.210	1.140
Monocalcium phosphate	0.250	0.190	0.120
Vitamin premix^1^	0.150	0.125	0.100
Se premix^2^	0.050	0.050	0.050
Phytase^3^	0.100	0.100	0.100
Salt	0.300	0.300	0.300
Lysine-HCl	0.380	0.370	0.350
dl-Methionine	0.050	0.030	0.010
l-Threonine	0.100	0.100	0.095
l-Tryptophan	0.025	0.030	0.030
Treatment premixes^4^	0.200	0.200	0.200
Total	100.0	100.0	100.0
Basal ingredient Zn, mg/kg	23.12	22.13	21.25
Calculated analysis			
ME, Kcal/kg	3,445.8	3,454.2	3,462.1
NE, Kcal/kg	2,635.5	2,658.0	2,678.7
CP, %	15.050	13.800	12.670
Fat, %	6.488	6.556	6.619
Total lysine, %	0.944	0.852	0.759
SID Lys, %	0.810	0.725	0.641
SID Lys:ME	2.351	2.100	1.851
Ca, %	0.601	0.550	0.503
P, %	0.409	0.382	0.355
Avail. Phos., %	0.251	0.231	0.211
SID Met:Lys	33.67	32.39	32.51
SID M + C:Lys	60.58	61.25	62.54
SID Thr:Lys	64.13	65.74	67.70
SID Tryp:Lys	18.13	18.52	18.53
SID Iso:Lys	58.99	58.65	59.06
SID Val:Lys	70.12	71.31	73.62

^1^Diet Phase GF4 vitamin premix provided the following per kilogram of diet; vitamin A, 3,968.3 IU; vitamin D3, 396.8 IU; vitamin E, 26.5 IU; vitamin K, 1.3 mg; vitamin B_12_, 0.02 mg; riboflavin, 5.3 mg; pantothenic acid, 13.2 mg; niacin, 19.8 mg. GF5 vitamin premix provided the following per kilogram of diet; vitamin A, 3,306.9 IU; vitamin D3, 330.7 IU; vitamin K, 1.1 mg; vitamin B_12_, 0.02 mg; riboflavin, 4.4 mg; pantothenic acid, 11 mg; niacin, 16.5 mg. GF6 provided the following per kilogram of diet; vitamin A, 2,645.5 IU; vitamin D3, 254.6 IU; vitamin E, 17.6 IU; vitamin K, 0.9 mg; vitamin B_12_, 0.02 mg; riboflavin, 3.5 mg; pantothenic acid, 8.8 mg; niacin, 13.2 mg.

^2^Se premix provided 0.3 mg Se per kilogram of diet.

^3^Phytase activity level 600 FTU/kg (Phyzyme, Danisco Animal Nutrition, Marlborough, UK).

^4^Dietary supplemental zinc sources were added to a fine ground corn premix to allow a constant formulation to be used during each growth phase (GF4, GF5, and GF6). The basal diet trace mineral premix for diets 1, 2, 4, 5, and 6 was added at 0.052% of the 0.20% which supplied 50 mg/kg of available Zn from ZnO. To this basal trace mineral, an additional 80 mg/kg of available Zn was added from ZnO for diet 2. For diets 4, 5, and 6, an additional 40, 60, and 80 mg/kg of available Zn was supplied from an organic source (Availa Zn 120 premix; 12% Zn; Zinpro Corporation, Eden Prairie, MN), respectively. For diet 3, a separate trace mineral premix was made to replace the 50 mg/kg Zn from ZnO with organic Zn. For all treatments, the trace mineral premixes also supplied per kg of diet: 50 mg Fe, 6.2 mg Mn, 4.66 mg Cu, and 0.19 mg I.

**Table 2.** Dietary analysis of elemental Zn content

Treatment	Diet phase, mg/kg			Target Zn content, mg/kg
	GF4	GF5	GF6	
Purdue University analysis^1^				
1	114.2	101.3	108.4	50
2	177.1	178.6	191.3	130
3	92.4	84.8	98.8	50
4	125.7	120.1	163.1	90
5	144.6	164.1	181.3	110
6	178.3	187.1	206.5	130
University of Missouri analysis^2^				
1	92.8	106	108	50
2	172	193	196	130
3	42.6	62.8	115	50
4	109	84.8	120	90
5	137	136	210	110
6	138	135	314	130
	ZnO	AvailZn120	PurduePMX	AvailaZnPMX
w/w%^3^	77.95	12.49	15.30	5.17

^1^Conducted in duplicate.

^2^Conducted in triplicate.

^3^Grams per 100 g of sample.

Diets were ground through a 1-mm screen in a Wiley Mill (Thomas Scientific, Swedesboro, NJ) prior to analysis. Percent dry matter and ash were analyzed by weighing a predried crucible and sample on the same scale after being dried in the drying oven for 12 h at 60 °C (Blue M Electric Company, Blue Island, IL), the sample was then ashed in the ashing oven for 8 h at 500 °C (Thermolyne 6000 muffle furnace, Dubuque, IA). Diet gross energy concentrations were determined using bomb calorimetry (Parr Isoperibol 6200 Calorimeter, Moline, IL). Diet nitrogen concentrations were determined using an automated nitrogen analyzer (LECO TruMac N, LECO, St. Joseph, MI, AOAC#990.03). Phosphorus was analyzed on the diet ash by the photometric analysis for phosphorus in animal feed method (AOAC#956.17, 1990, Official Methods of Analysis, 15th ed.) using a BioTek Epoch Plate Reader (BioTek Instruments, Inc., Winooski, VT) at a wavelength of 405 nm. Diet Zn concentration was determined by atomic absorption spectrophotometry (Spectra AA 220FS, Varian) following nitric-perchloric digestion. Assays conducted at Purdue University were conducted in duplicate with an internal standard and all values were completed with less than a CV of 5% and were adjusted to the internal standard recovery for each assay. Zinc analysis was conducted at both Purdue University and University of Missouri due to the variable nature of Zn assays.

### Animal Growth and Performance

All pigs and feeders were weighed at the beginning and end of each growth phase and acute heat event. Pen averages were determined for BW, ADG, ADFI, and G:F for each growth phase, heat event, and overall. Cumulative water intake was recorded at each weigh day through two water meters (1700 model, iSTEC Corporation, Sparta NJ) per room.

### Live Ultrasound Scanning

On day 63 of the study, all pigs were scanned with a live ultrasound (Aloka 500; Hitachi Aloka Medical Ltd., Tokoyo, Japan) to measure backfat (BF) depth at the last (LR) and 10th rib (TR) in addition to loin muscle depth and loin muscle area (**LMA**). Pigs in the HS environment were scanned on day 70 following the third heat wave for the same measurements. The LR, TR, and LMA were used with BW to determine the calculated percent lean and were analyzed as pen averages. The description of the percent lean equation can be found in Table 3 (Hicks et al., 1998).

**Table 3.** Interactive effects of diet and environment on live pig body composition using real-time ultrasound

Environment	TN						HS						SE	Treatment^1^, *P*<
Diets 1 to 6, ZnO/Organic Zn, mg/kg^3^	50/0	130/0	0/50	50/40	50/60	50/80	50/0	130/0	0/50	50/40	50/60	50/80		
LR, BF, mm	16.28	16.97	16.96	17.14	16.85	17.20	15.72	16.40	15.73	16.40	16.69	15.98	0.088	0.56
TR, BF, mm	17.44	17.78	18.24	18.39	17.83	18.30	16.32	17.10	16.69	17.00	17.53	16.74	0.086	0.21
Loin muscle depth, cm	5.61	5.63	5.55	5.59	5.49	5.53	5.55	5.40	5.55	5.33	5.58	5.31	0.093	0.08
LMA, cm^2^	54.41	56.14	55.41	55.12	54.49	55.97	54.49	52.95	53.07	52.60	54.57	52.75	1.074	0.15
BW, day 63, kg	126.9	129.3	127.4	128.5	127.7	130.3	124.5	125.2	123.0	123.0	125.7	122.7	3.141	0.14
Calculated % lean^2^	54.32	54.23	54.27	53.89	53.98	53.85	55.06	54.02	54.80	54.36	54.45	54.68	0.751	0.69

^1^Effect of overall 12 treatment combinations.

^2^Calculated % lean equation: ((11.08 + (0.218 × BW) + (−3.31 × BF LR) + (0.346 × LMA) + (−0.715 × BF TR))/(BW × 0.74)) × 100 (Hicks et al., 1998).

^3^Dietary available zinc supplementation: (1) 50 mg/kg ZnO; (2) 130 mg/kg ZnO: (3) 50 mg/kg of organic Zn (Availa Zn); (4) 50 mg/kg ZnO + 40 mg/kg organic Zn); (5) 50 mg/kg ZnO + 60 mg/kg organic Zn; and (6) 50 mg/kg ZnO + 80 mg/kg organic Zn.

### Statistical Analysis

Pen was used as the experimental unit (*n* = 120) for all growth performance and live ultrasound data. All data were analyzed using the MIXED procedure of SAS 9.4 (SAS Institute Inc., Cary, NC) with treatment as a fixed effect and a replicate as a random effect. Data were analyzed as a series of preplanned contrasts for the effect of environment (HS vs. TN), Zn source (inorganic vs. organic), Zn level (50 vs. 90 vs. 110 vs. 130), and their two-way and three-way interactions. Values of *P* ≤ 0.05 were considered significant and 0.05 < *P* ≤ 0.10 were considered trends.

## Results

### Growth Performance

For the first 18 d of the study, there was no difference between HS and TN treatment in ADG, ADFI, or G:F (*P* > 0.15; Table 4). During, the initial three-day HS acclimation period (days 18 to 21), increased temperatures led to a reduction in feed intake in HS animals (*P* < 0.05). During the first heat event (days 21 to 24), HS pigs had a lower ADG (*P* < 0.05), ADFI (*P* < 0.05), and G:F (*P* < 0.05). The effects from the initial heat event in the HS environment were seen over the next 18-d period (days 24 to 42) where ADG (*P* < 0.05), ADFI (*P* < 0.05), and day 42 BW (*P* < 0.05) were reduced (*P* < 0.05). However, the HS pigs had increased G:F compared to their TN counterparts (*P* < 0.05). The second heat wave (days 42 to 45), which had the highest recorded average humidity during the study, reduced ADG (*P* < 0.05), ADFI (*P* < 0.05), G:F (*P* < 0.05), and day 45 BW by almost 3 kg in HS pigs (*P* < 0.05). Days 45 to 63 showed another decrease in HS ADG and ADFI (*P* < 0.05) with an increase in G:F (*P* < 0.05). By the time of the last growth comparison timepoint (day 63), HS reduced ADG and ADFI overall and final BW from 128.4 kg in TN to 124.0 kg (*P* < 0.05). When evaluating growth performance between growth phases, there were no differences in ADG, ADFI, or efficiency in GF4 period (*P* > 0.1). During GF5 and GF6 periods, ADG (*P* < 0.05) and feed intake (*P* < 0.05) were reduced by HS, but no differences between HS and TN in G:F for GF5 or GF6 production phases.

**Table 4.** Main effect of environmental temperature on grow-finish pig growth performance

Environment^1^	TN	HS	SE	Environment, *P*<
Initial BW, kg	71.97	72.38	0.906	0.50
Days 0 to 18				
ADG, kg/d	0.897	0.897	0.031	0.98
ADFI, kg/d	2.699	2.725	0.075	0.64
G:F	0.331	0.325	0.005	0.40
Day 18 BW, kg	88.12	88.41	1.375	0.71
Days 18 to 21				
ADG, kg/d	0.974	0.946	0.043	0.58
ADFI, kg/d	2.888	2.667	0.051	<0.05
G:F	0.334	0.360	0.012	0.11
Day 21 BW, kg	91.08	91.24	1.318	0.83
Days 21 to 24				
ADG, kg/d	0.991	0.655	0.037	<0.05
ADFI, kg/d	3.235	2.628	0.037	<0.05
G:F	0.306	0.268	0.013	<0.05
Day 24 BW, kg	94.05	93.19	1.246	0.23
Days 24 to 42				
ADG, kg/d	0.920	0.856	0.016	<0.05
ADFI, kg/d	3.163	2.829	0.072	<0.05
G:F	0.290	0.302	0.005	<0.05
Day 42 BW, kg	110.4	108.40	1.505	<0.05
Days 42 to 45				
ADG, kg/d	0.920	0.732	0.032	<0.05
ADFI, kg/d	3.147	2.665	0.065	<0.05
G:F	0.312	0.284	0.015	<0.05
Day 45 BW, kg	113.3	110.62	1.571	<0.05
Days 45 to 63				
ADG, kg/d	0.859	0.760	0.016	<0.05
ADFI, kg/d	3.197	2.705	0.039	<0.05
G:F	0.268	0.281	0.003	<0.05
Day 63 BW, kg	128.4	124.03	1.971	<0.05
Diet phases				
Days 0 to 21 (GF4)				
ADG, kg/d	0.908	0.896	0.026	0.63
ADFI, kg/d	2.733	2.714	0.071	0.72
G:F	0.332	0.330	0.003	0.71
Days 21 to 42 (GF5)				
ADG, kg/d	0.930	0.828	0.016	<0.05
ADFI, kg/d	3.177	2.800	0.062	<0.05
G:F	0.291	0.295	0.005	0.48
Days 42 to 63 (GF6)				
ADG, kg/d	0.872	0.757	0.016	<0.05
ADFI, kg/d	3.189	2.699	0.041	<0.05
G:F	0.273	0.280	0.003	0.13
Overall				
Days 0 to 63				
ADG, kg/d	0.901	0.825	0.014	<0.05
ADFI, kg/d	3.033	2.739	0.052	<0.05
G:F	0.297	0.301	0.002	0.12
HS pigs only				
Days 63 to 6				
ADG, kg/d	—	0.660	0.093	—
ADFI, kg/d	—	2.476	0.106	—
G:F	—	0.267	0.038	—
Day 63 BW, kg^2^	—	124.0	3.192	—
Day 66 BW, kg	—	125.9	3.112	—
Days 66 to 70				
ADG, kg/d	—	0.779	0.067	—
ADFI, kg/d	—	2.819	0.125	—
G:F	—	0.277	0.021	—
Days 63 to 70				
ADG, kg/d	—	0.729	0.049	—
ADFI, kg/d	—	2.666	0.098	—
G:F	—	0.273	0.020	—
Day 70 BW, kg	—	129.3	3.090	—
Days 0 to 70				
ADG, kg/d	—	0.817	0.024	—
ADFI, kg/d	—	2.726	0.068	—
G:F	—	0.299	0.005	—

^1^Environments were TN and cyclic heat (HS).

^2^Day 63 pen mean weight after removal of 1 pig/pen for carcass data at the Purdue University meats lab. Now 4 pigs/pen.

From days 0 to 63, three-way interactions between Zn source, Zn level, and environment were evident for ADG (*P* = 0.05) and ADFI (*P* < 0.05; Table 5; Supplementary Table S6). Under TN conditions, diet 6 had the highest ADG (0.936 kg/d) closely followed by diet 2 (0.912 kg/d; Table 5). However, when under HS conditions, diet 6 had the lowest ADG (0.802 kg/d) and feed intake (2.788 kg/d) when compared to the rest of the diets while diet 5 had the highest ADG (0.850 kg/d) again followed closely by diet 2 (0.849 kg/d). This ADG interaction was driven by an identical interaction in overall (days 0 to 63) ADFI. A similar three-way interaction was observed during the first (ADG *P* < 0.05; ADFI *P* < 0.05) and second heat event (ADG *P* < 0.05; ADFI *P* < 0.05) with no differences in G:F (*P* > 0.1). During GF5, pigs fed diet 2 (0.945 kg/d) and diet 6 (0.964 kg/d) had the highest ADG under TN conditions whereas diet 5 had the greatest ADG (0.872 kg/d) closely followed by diet 2 (0.849 kg/d) under HS (*P* < 0.05; Table 5). Under HS conditions, diet 2 and diet 5 also had the greatest ADFI when compared to the other diets and a three-way interaction was observed for ADFI during this production phase (*P* < 0.05; Table 5). A similar pattern was observed during GF6 where another three-way interaction was observed for ADG (*P* < 0.05) and ADFI (*P* < 0.05; Table 5). Pigs undergoing HS had the highest ADG when fed diet 2 (0.802 kg/d), closely followed by diet 5 (0.793 kg/d) and both diets had the highest ADFI when compared to the rest of the dietary treatments (*P* < 0.05). Under TN conditions, diet 6 produced the highest ADG (0.927 kg/d) and ADFI. During the last heat event (days 63 to 66), pigs fed diets 1 and 3 had the greatest ADG (Supplementary Tables S5 and S7). Diet 4 in the HS environment had the greatest (*P* < 0.05) ADG from days 66 to 70 with the other dietary treatments having very similar ADG (Supplementary Table S5). Diet 1 had the greatest ADFI from days 66 to 70 under HS.

**Table 5.** Interactive means of diet by environment on grow-finish pig growth performance

Environment	TN (days 0 to 63)						HS (days 0 to 70)						SE	*P*-value	
Diet^1^	1	2	3	4	5	6	7	8	9	10	11	12		Treatment^2^	Environment
ZnO/Organic diet Zn, mg/kg	50/0	130/0	0/50	50/40	50/60	50/80	50/0	130/0	0/50	50/40	50/60	50/80			
Initial no. pens	10	10	10	10	10	10	10	10	10	10	10	10	—		
Initial no. pigs	50	50	50	50	50	50	50	50	50	50	50	50	—		
Initial BW, kg	71.92	72.25	72.05	71.68	72.22	71.69	72.55	72.05	71.96	72.73	72.46	72.49	1.938	0.63	0.50
Days 0 to 18															
ADG, kg/d	0.836	0.924	0.890	0.891	0.910	0.933	0.871	0.948	0.883	0.894	0.886	0.853	0.974	0.49	0.98
ADFI, kg/d	2.624	2.726	2.690	2.617	2.785	2.751	2.752	2.794	2.631	2.750	2.731	2.693	0.117	0.21	0.64
G:F	0.315	0.338	0.328	0.338	0.325	0.339	0.315	0.340	0.333	0.323	0.322	0.318	0.013	0.74	0.40
Day 18 BW, kg	86.96	88.89	88.08	87.72	88.60	88.49	88.23	88.56	87.86	88.83	88.40	88.59	2.458	0.63	0.71
Days 18 to 21															
ADG, kg/d	0.994	0.987	1.051	0.937	1.049	0.827	0.994	0.899	0.942	0.973	0.883	1.014	0.094	0.35	0.58
ADFI, kg/d	2.813	2.938	2.976	2.811	2.908	2.884	2.718	2.764	2.591	2.746	2.642	2.538	0.109	0.56	<0.05
G:F	0.352	0.315	0.353	0.312	0.367	0.306	0.353	0.340	0.368	0.342	0.360	0.398	0.029	0.95	0.11
Day 21 BW, kg	89.94	91.89	91.23	90.53	91.94	90.97	91.48	90.82	91.00	91.44	91.05	91.69	2.525	0.94	0.83
Days 21 to 24															
ADG, kg/d^3^	0.918^a^	0.977^a^	0.940^a^	0.995^ad^	0.973^a^	1.144^af^	0.524^b^	0.811^acg^	0.630^bc^	0.611^bc^	0.730^abceg^	0.621^bc^	0.094	<0.05	<0.05
ADFI, kg/d	3.025^a^	3.182^ab^	3.184^ab^	3.289^b^	3.400^b^	3.330^b^	2.644^c^	2.728^c^	2.559^c^	2.700^c^	2.630^c^	2.506^c^	0.090	<0.05	<0.05
G:F	0.300	0.307	0.297	0.304	0.286	0.342	0.213	0.303	0.265	0.274	0.275	0.271	0.033	0.45	<0.05
Day 24 BW, kg	92.70	94.82	94.05	93.51	94.86	94.40	93.05	93.64	92.60	93.27	93.22	93.38	0.040	0.74	0.23
Days 24 to 42															
ADG, kg/d	0.894	0.939	0.910	0.911	0.930	0.934	0.859	0.834	0.838	0.841	0.898	0.868	0.040	0.10	<0.05
ADFI, kg/d	3.082^a^	3.114^a^	3.215^ac^	3.278^ae^	3.062^ag^	3.230^ah^	2.794^b^	2.904^abdfi^	2.771^b^	2.776^b^	2.891^abdfi^	2.837^bg^	0.107	<0.05	<0.05
G:F	0.291	0.303	0.281	0.278	0.296	0.288	0.306	0.287	0.301	0.303	0.311	0.305	0.010	0.48	<0.05
Day 42 BW, kg	108.8	111.7	110.4	110.0	110.4	111.2	108.5	108.7	107.6	108.4	109.3	107.9	2.837	0.45	<0.05
Days 42 to 45															
ADG, kg/d	1.053^a^	0.913^abc^	0.814^b^	0.950^ae^	0.832^b^	0.953^a^	0.750^be^	0.880^ab^	0.735^bd^	0.730^bd^	0.640^bd^	0.659^bd^	0.080	<0.05	<0.05
ADFI, kg/d	3.163^a^	3.162^a^	3.068^ac^	3.210^ae^	3.021^ad^	3.255^ag^	2.537^b^	2.683^bcd^	2.502^b^	2.757^abfh^	2.794^ab^	2.720^bcdh^	0.160	<0.05	<0.05
G:F	0.332	0.290	0.309	0.324	0.277	0.339	0.287	0.324	0.296	0.283	0.238	0.275	0.027	0.65	<0.05
Day 45 BW, kg	112.0	114.5	113.1	113.1	112.9	114.1	110.5	111.3	109.8	110.6	111.2	110.3	2.806	0.24	<0.05
Days 45 to 63															
ADG, kg/d	0.847	0.844	0.830	0.873	0.838	0.922	0.794	0.790	0.751	0.704	0.819	0.705	0.039	0.12	<0.05
ADFI, kg/d	3.140^a^	3.140^a^	3.144^a^	3.210^a^	3.228^a^	3.327^a^	2.701^b^	2.722^b^	2.680^b^	2.616^b^	2.860^b^	2.649^b^	0.096	<0.05	<0.05
G:F	0.270^a^	0.268^ac^	0.263^ae^	0.272^a^	0.258^ag^	0.275^a^	0.294^b^	0.290^adfh^	0.278^a^	0.268^a^	0.286^af^	0.266^a^	0.008	<0.05	<0.05
Day 63 BW, kg	126.9	129.3^a^	127.8^a^	128.5^a^	127.7^a^	130.3^a^	124.5	125.2	123.1	123.0	125.7	122.7	3.099	0.14	<0.05
Diet phases															
Days 0 to 21 (GF4)															
ADG, kg/d	0.858	0.934	0.913	0.898	0.925	0.918	0.901	0.893	0.907	0.891	0.885	0.901	0.046	0.78	0.63
ADFI, kg/d	2.651	2.777	2.731	2.667	2.802	2.770	2.736	2.790	2.625	2.747	2.718	2.671	0.108	0.39	0.72
G:F	0.322	0.336	0.333	0.335	0.331	0.331	0.330	0.327	0.345	0.324	0.324	0.330	0.009	0.27	0.71
Days 21 to 42 (GF5)															
ADG, kg/d	0.898^a^	0.945^ab^	0.914^ad^	0.926^af^	0.932^ah^	0.964^aj^	0.811^acgik^	0.849^ak^	0.795^acegik^	0.808^acegik^	0.872^a^	0.833^ack^	0.040	<0.05	<0.05
ADFI, kg/d	3.074^a^	3.124^a^	3.210^ac^	3.278^ae^	3.100^ag^	3.265^ai^	2.773^b^	2.879^adfhj^	2.742^b^	2.766^b^	2.854^adfhj^	2.790^b^	0.097	<0.05	<0.05
G:F	0.292	0.303	0.283	0.283	0.292	0.294	0.292	0.296	0.288	0.292	0.306	0.298	0.010	0.82	0.48
Days 42 to 63 (GF6)															
ADG, kg/d	0.877^a^	0.854^ac^	0.840^ad^	0.898^ae^	0.838^a^	0.927^ag^	0.774^afh^	0.802^ah^	0.750^bcdh^	0.708^bh^	0.793^ah^	0.715^bh^	0.039	<0.05	<0.05
ADFI, kg/d	3.139^a^	3.139^a^	3.132^a^	3.210^a^	3.198^a^	3.316^a^	2.677^b^	2.716^b^	2.653^b^	2.636^b^	2.850^b^	2.659^b^	0.100	<0.05	<0.05
G:F	0.280	0.271	0.267	0.28	0.261	0.279	0.289	0.295	0.280	0.267	0.278	0.269	0.007	0.19	0.13
Overall															
Days 0 to 63															
ADG, kg/d	0.878^a^	0.912^ac^	0.890^a^	0.908^ae^	0.884^a^	0.936^ag^	0.830^adfh^	0.849^ah^	0.817^adfh^	0.803^b^	0.850^ah^	0.802^b^	0.029	0.05	<0.05
ADFI, kg/d	2.955^a^	3.013^ac^	3.025^ae^	3.050^af^	3.039^ah^	3.117^aj^	2.730^b^	2.797^adgik^	2.673^b^	2.718^b^	2.807^adgik^	2.708^adgik^	0.080	<0.05	<0.05
G:F	0.297	0.303	0.294	0.297	0.291	0.300	0.304	0.303	0.305	0.295	0.303	0.296	0.005	0.52	0.12

^1^Effect of overall 12 treatment combinations.

^2^Dietary available zinc supplementation: (1) 50 mg/kg ZnO, (2) 130 mg/kg ZnO, (3) 50 mg/kg of organic Zn (Availa Zn), (4) 50 mg/kg ZnO + 40 mg/kg organic Zn), (5) 50 mg/kg ZnO + 60 mg/kg organic Zn, and (6) 50 mg/kg ZnO + 80 mg/kg organic Zn.

^3^Superscripts indicate means comparison differences (P<.05). Any value with shared superscript letters are not different from each other at P<0.05).

### Live Ultrasound Characteristics

On day 63, pigs exposed to HS had decreased BF at the LR (*P* < 0.05) and TR (*P* < 0.05), loin muscle depth (*P* < 0.05), LMA (*P* < 0.05), and a tendency to be leaner (*P* = 0.10; Table 6). A tendency for a diet and environment interaction was noted for loin muscle depth (*P* = 0.08; Table 3; Supplemental Table S8). Under TN conditions, pigs fed diet 2 had the largest LMA (56.14 cm^2^) which was closely followed by diet 6 (55.97 cm^2^). However, under HS conditions, LMA was smaller in pigs fed diets 6 (52.75 cm^2^) and 2 (53.07 cm^2^) relative to the other diets, and diet 5 produced the largest LMA under HS conditions (54.57 cm^2^). When HS pigs were scanned on day 70, there were no differences observed between diets (*P* > 0.10).

**Table 6.** Main effect of environment on live pig body composition using real-time ultrasound

Environment^1^	TN^2^	HS	SE	*P*
BF, LR, mm	16.90	16.15	0.073	<0.05
BF, TR, mm	17.99	16.90	0.066	<0.05
Loin muscle depth, cm	5.570	5.450	0.043	<0.05
LMA, cm^2^	55.26	53.40	0.606	<0.05
BW day 63, kg	128.4	124.0	2.488	<0.05
Calculated % lean^3^	54.09	54.56	0.584	0.10

^1^Environments were TN and cyclic heat (HS).

^2^Pen used as experimental unit (TN *n* = 60; HS *n* = 60).

^3^Calculated % lean equation: ((11.08 + (0.218 × BW) + (−3.31 × BF LR) + (0.346 × LMA) + (−0.715 × BF TR))/(BW × 0.74)) × 100 (Hicks et al., 1998).

### Water Intake

From days 10 to 18, there were no differences (*P* = 0.43; Table 7) in water intake between TN and HS groups, which was expected since the HS rooms were kept at the same temperature to this date (Figure 2). During the brief heat acclimation period before the first heat event (days 18 to 21), no differences were observed, but HS began to numerically increase water intake by 2 L/d/pig (Table 7). During days 21 to 24, 24 to 42, 42 to 45, and 45 to 63, HS pigs utilized 50%+ more water than TN (*P* < 0.05) group (Table 7). As expected, no differences were observed for GF4 (*P* = 0.30). However, during GF5 and GF6 HS conditions increased water intake by 7.3 and 6.3 L/d/pig, respectively (*P* < 0.05; Table 7). From days 63 to 70, water intake remained high for HS pigs and was similar to the water intake in GF5 and GF6 (Table 7). Overall, days 10 to 63, pigs under HS utilized 6 L/d/pig more water than TN pigs (*P* < 0.05; Table 7).

**Table 7.** Effect of environment on water intake

Environment^1^	TN, liters/d/pig	HS, liters/d/pig	SE	*P*-value
Days 10 to 18^2^	5.05	7.55	2.05	0.44
Days 18 to 21^3^	6.78	8.83	1.17	0.23
Days 21 to 24^4^	7.90	16.6	1.65	<0.05
Days 24 to 42	7.61	14.6	1.19	<0.05
Days 42 to 45^5^	6.17	15.6	0.99	<0.05
Days 45 to 63	6.36	12.0	1.06	<0.05
Diet phases				
Days 10 to 21 (GF4)	6.32	8.02	1.11	0.30
Days 21 to 42 (GF5)	7.64	14.9	1.20	<0.05
Days 42 to 63 (GF6)	6.17	12.5	1.02	<0.05
Overall				
Days 0 to 63	6.85	13.0	1.04	<0.05
Cyclic heat pigs only				
Days 63 to 66^6^	—	12.3	2.50	—
Days 66 to 70	—	14.3	1.33	—
Days 63 to 70	—	13.7	1.62	—
Days 0 to 70	—	12.7	1.28	—

^1^Environments were TN and cyclic heat (HS).

^2^Data collection on water intake began on day 10 of the study.

^3^Acclimation period leading up to first heat event.

^4^First heat event.

^5^Second heat event. Day 63 removal of 1 pig/pen for carcass data at the Purdue University meats lab.

^6^Third heat event.

## Discussion

The present study was designed to determine the effect of supplemental zinc source and level on growth performance and estimated carcass composition in finishing pigs undergoing chronic cyclical heat stress. Heat stress substantially influences an animal’s physiology. In an attempt to maintain euthermia, swine reduce feed intake, which with sustained exposure to elevated temperature can result in decreased growth rate and G:F (Ross et al., 2015). Although decreased feed intake affects body composition and growth rate, nutrient restriction from heat stress also affects intestinal barrier function and morphology (Ferraris and Carey, 2000).

Zinc is involved with most metabolic interactions and functions as a cofactor to over 300 enzymes within the body, and is important for protein synthesis, enterocyte regeneration, and intestinal barrier function (Alam et al., 1994). Weanling pigs’ growth rate responds quadratically to increasing pharmacological levels of inorganic zinc (Hill et al., 2001). Moreover, the source of Zn can affect swine’s HS response due to its variation in bioavailability with organic sources being more available to the pig. Weanling pigs fed chelated zinc have been shown to have greater concentrations of zinc within their serum and longissimus dorsi when compared to weanling pigs fed ZnO (Wang et al., 2010). Pigs fed ZnO also had a greatly increased Zn fecal concentration compared to the chelated Zn treatment, demonstrating that organic Zn is much more available to the pig. Finishing pigs supplemented with 120 mg/kg inorganic/organic Zn lost less weight than their 120 mg/kg inorganic Zn counterparts when undergoing a 12-h HS event, but consumed the same amount of feed (Pearce et al., 2015). Interestingly, Zn concentrations within the ileum and colon did not differ between dietary and environmental treatments (Pearce et al., 2015). Other studies have shown that increasing dietary Zn levels increase Zn concentrations within serum, bone, liver, and other organs, but Zn source does not impact Zn concentrations within these locations of the body (Revy et al., 2004; Shinde et al., 2006). In the present study, ADG and ADFI were greatest under TN conditions when pigs were fed 130 mg/kg inorganic Zn and 130 mg/kg inorganic/organic Zn blend. However, during acute heat events and elevated environmental temperatures ADG and ADFI were greatest when pigs were fed 130 mg/kg inorganic Zn and the 110 mg/kg inorganic/organic Zn blend and decreased when fed the 130 mg/kg inorganic/organic Zn blend. Our 110 mg/kg inorganic/organic Zn blend had a similar amount of organic Zn supplemented to Pearce et al.’s (2015) 120 mg/kg inorganic/organic Zn blend where both diets contained 60 mg/kg organic zinc. This may indicate that a level of near 60 mg/kg may be optimal in terms of supplemental organic zinc levels when in combination with inorganic zinc, while 80 mg/kg organic zinc and a total of 130 mg/kg Zn is too much for the body to efficiently handle under these HS conditions. However, another possibility that should be explored is whether the level of phytase inclusion would improve the bioavailability even further. Water intake was also increased under HS conditions which is indicative of increasing temperatures as pigs will drink the water to cool down, but also spray themselves with the water in order to increase evaporative cooling.

In the present study, pigs exposed to HS were leaner than their TN counterparts, however, HS pigs were smaller on days 63 than TN pigs (124.11 vs. 128.34 kg) and thus had a smaller amount of fat relative to muscle. Heat stress is known for its effects on changing nutrient partitioning and changing body composition to emphasize lipid accretion over protein accretion (Close et al., 1971; Verstegen et al., 1978; Heath, 1983; Bridges et al., 1998; Collin et al., 2001). However, under our experimental conditions, this was not the case, as we observed both reduced fat and muscle composition of the loin which was likely due to our HS pigs being smaller than their TN counterparts on day 63. A study conducted in 1988 reported that heat stress has a greater impact in heavier pigs on carcass composition than smaller pigs; that study, reported heavier pigs had increased fat deposition and experienced changes associated with lipid metabolism when compared to their smaller counterparts (Christon, 1988). The fact that our HS pigs were smaller at day 63 when the live ultrasound scans were performed may partially explain why they appeared leaner than our TN pigs. Although BW was highly significant on day 63 between the TN pigs and HS pigs, when day 63 BW was used as a covariate it did not remove significance and trends observed in the contrasts prior to running BW as a covariate for body composition estimates.

It is interesting to consider the pigs’ adaptive response to the heat stress from the acute heat events and then summer-like heat temperatures following them. During the first acute heat event, days 21 to 24, ADG decreased by 34%, ADFI decreased by 19%, and G:F decreased by 12%. In the following summer-like conditions during days 24 to 42, ADG (7%) and ADFI (10.6%) decreased but G:F increased by 4%. During the next acute heat event, days 42 to 45, ADG (20%), ADFI (15%), and G:F (9%) all decreased. During the last elevated temperature period during days 45 to 63, ADG (11.5%) and ADFI (15%) decreased but G:F increased by 5%. It would appear that the first acute heat wave had a greater impact on the growth rate than the second acute heat wave, indicating some possible adaptation. However, counter to this thought is the indication that the pigs did not recover as well from the second acute heat event during the second extended elevated temperature period with greater reductions in ADG and ADFI in this second postacute heat event period. It is also curious that the G:F improved during the postacute heat event periods despite elevated temperatures (days 24 to 42 and 45 to 63). This may be related to repaired and improved gut function after the acute heat stress periods or it may also be related to the reduced fat depth and deposition for pigs under HS, as it takes substantially more energy and is less efficient to deposit fat than lean and the lower ADFI under HS may not have provided the needed energy for similar fat deposition rates as TN pigs.

## Conclusions

Under TN conditions, feeding available zinc at 130 mg/kg from an inorganic source and from a 50 inorganic/80 organic mg/kg zinc blend increased growth rate which was largely driven by an increase in feed intake. Ultrasonically measured LMA was similar in size between both the inorganic/organic blend and inorganic sourced zinc at 130 mg/kg. Under HS conditions, pigs grew 8% slower and ate less overall. Pigs in the HS environment supplemented with the 50 inorganic/60 organic mg/kg blend or the inorganic zinc at 130 mg/kg had greater feed intake and greatest growth rate when compared to HS animals supplemented with the other zinc concentrations and source blends. The HS environment also decreased LMA but also TR BF which resulted in a greater percent lean. The negative interaction between the 50 inorganic/80 organic mg/kg blend in the HS environment on growth performance is due to an unclear mechanism and warrants future research. Available Zn supplementation levels above the NRC (2012) 50 mg/kg guideline appear to provide greater growth performance under both TN and HS conditions.

## Supplementary Material

txae029_suppl_Supplementary_Tables

## References

[CIT0001] Acda, S. P., and B. J. Chae. 2002. A review on the applications of organic trace minerals in pig nutrition. Pak. J. Nutr. 1:25–30. doi:10.3923/pjn.2002.25.30

[CIT0002] Alam, A. N., S. A. Sarker, M. A. Wahed, M. Khatun, and M. M. Rahaman. 1994. Enteric protein loss and intestinal permeability changes in children during acute shigellosis and after recovery: effect of zinc supplementation. Gut. 35:1707–1711. doi:10.1136/gut.35.12.1707 7829006 PMC1375257

[CIT0003] Baumgard, L. H., and R. P. Rhoads, Jr. 2013. Effects of heat stress on postabsorptive metabolism and energetics. Annu. Rev. Anim. Biosci. 1:311–337. doi:10.1146/annurev-animal-031412-103644 25387022

[CIT0004] Bridges, T., L. W. Turner, and R. S. Gates. 1998. Economic evaluation of misting-cooling systems for growing/finishing swine through modeling. Appl. Eng. Agric. 14:425–430. doi:10.13031/2013.19398

[CIT0005] Christon, R. 1988. The effect of tropical ambient temperature on growth and metabolism in pigs. J. Anim. Sci. 66:3112–3123. doi:10.2527/jas1988.66123112x 3230073

[CIT0006] Close, W. H., L. E. Mount, and I. B. Start. 1971. The influence of environmental temperature and plane of nutrition on heat losses from groups of growing pigs. Anim. Sci. 13:285–294. doi:10.1017/s000335610002972x

[CIT0007] Collin, A., J. van Milgen, S. Dubois, and J. Noblet. 2001. Effect of high temperature and feeding level on energy utilization in piglets. J. Anim. Sci. 79:1849–1857. doi:10.2527/2001.7971849x 11465372

[CIT0008] Dardenne, M. 2002. Zinc and immune function. Eur. J. Clin. Nutr. 56:S20–S23. doi:10.1038/sj.ejcn.1601479 12142956

[CIT0009] DeShazer, J., G. LeRoy Hahn, and H. Xin. 2009. Chapter 1: Basic principles of the thermal environment and livestock energetics. In: Livestock energetics and thermal environmental management. American Society of Agricultural and Biological Engineers, St. Joseph, Michigan; 1–22. doi:10.13031/2013.28294

[CIT0010] Feldpausch, J. 2019. Interactive effects of nutrition, environment, and processing on fresh pork quality, intestinal biomarkers of heat stress in swine, and career success factors for agricultural students [Dissertation]. Purdue University, West Lafayette, Indiana.

[CIT0011] Ferraris, R. P., and H. V. Carey. 2000. Intestinal transport during fasting and malnutrition. Annu. Rev. Nutr. 20:195–219. doi:10.1146/annurev.nutr.20.1.195 10940332

[CIT0012] Glover, C. N., N. R. Bury, and C. Hogstrand. 2003. Zinc uptake across the apical membrane of freshwater rainbow trout intestine is mediated by high affinity, low affinity, and histidine-facilitated pathways. Biochim. Biophys. Acta–. 1614:211–219. doi:10.1016/s0005-2736(03)00178-0 12896814

[CIT0013] Godfray, H. C. J., I. R. Crute, L. L. D. Haddad, J. F. Muir, N. Nisbett, J. Pretty, S. Robinson, T. Camilla, and W. Rosalind. 2010. The future of the global food system. Phil. Trans. R. Soc. B. 365:2769–2777. doi:10.1098/rstb.2010.0180 20713383 PMC2935131

[CIT0014] Heath, M. E. 1983. The effects of rearing-temperature on body composition in young pigs. Comp. Biochem. Physiol. A Comp. Physiol. 76:363–366. doi:10.1016/0300-9629(83)90338-9 6139210

[CIT0015] Hicks, C., A. P. Schinckel, J. C. Forrest, J. T. Akridge, J. R. Wagner, and W. Chen. 1998. Biases associated with genotype and sex in prediction of fat-free lean mass and carcass value in hogs. J. Anim. Sci. 76:2221–2234. doi:10.2527/1998.7692221x 9781476

[CIT0016] Hill, G. M., D. C. Mahan, S. D. Carter, G. L. Cromwell, R. C. Ewan, R. L. Harrold, A. J. Lewis, P. S. Miller, G. C. Shurson, and T. L. Veum; NCR-42 Committee on Swine Nutrition. 2001. Effect of pharmacological concentrations of zinc oxide with or without the inclusion of an antibacterial agent on nursery pig performance. J. Anim. Sci. 79:934–941. doi:10.2527/2001.794934x 11325200

[CIT0017] Kidd, M., P. Ferket, and J. Garlich. 1997. Nutritional and osmoregulatory functions of betaine. Worlds Poul. Sci. J. 53:125–139. doi:10.1079/wps19970013

[CIT0018] Lima, A. A., L. F. Brito, H. B. Ribeiro, M. C. Martins, A. P. Lustosa, E. M. Rocha, N. L. Lima, C. M. Monte, and R. L. Guerrant. 2005. Intestinal barrier function and weight gain in malnourished children taking glutamine supplemented enteral formula. J. Pediatr. Gastroenterol. Nutr. 40:28–35. doi:10.1097/00005176-200501000-00006 15625423

[CIT0019] Mayorga, E. J., D. Renaudeau, B. C. Ramirez, J. W. Ross, and L. H. Baumgard. 2019. Heat stress adaptations in pigs. Anim. Front. 9:54–61. doi:10.1093/af/vfy035 32002240 PMC6951998

[CIT0020] Moeckel, G. W., R. Shadman, J. M. Fogel, and S. M. Sadrzadeh. 2002. Organic osmolytes betaine, sorbitol and inositol are potent inhibitors of erythrocyte membrane ATPases. Life Sci. 71:2413–2424. doi:10.1016/s0024-3205(02)02035-0 12231402

[CIT0021] National. 2012. Nutrient Requirements of Swine: Eleventh Revised Edition. Washington, DC: National Academies Press. doi:10.17226/13298

[CIT0022] O’Dell, B. L. 1969. Effect of dietary components upon zinc availability123: a review with original data. Am. J. Clin. Nutr. 22:1315–1322. doi:10.1093/ajcn/22.10.1315 4981186

[CIT0023] Pearce, S., M. -V. Sanz Fernandez, J. Torrison, M. Wilson, L. Baumgard, and N. Gabler. 2015. Dietary organic zinc attenuates heat stress–induced changes in pig intestinal integrity and metabolism. J. Anim. Sci. 93:4702–4713. doi:10.2527/jas.2015-9018. 26523563

[CIT0024] Pollmann, D. 2010. Seasonal effects on sow herds: industry experience and management strategies. J. Anim. Sci. 88:9. (Abstr.)

[CIT0025] Revy, P. S., C. Jondreville, J. Y. Dourmad, and Y. Nys. 2004. Effect of zinc supplemented as either an organic or an inorganic source and of microbial phytase on zinc and other minerals utilisation by weanling pigs. Anim. Feed Sci. Technol. 116:93–112. doi:10.1016/j.anifeedsci.2004.04.003

[CIT0026] Ross, J., B. Hale, N. Gabler, R. Rhoads, A. Keating, and L. Baumgard. 2015. Physiological consequences of heat stress in pigs. Anim. Prod. Sci. 55:1381–1390. doi:10.1071/AN15267

[CIT0027] Schieck, S. 2019. Heat stress affects both grow-finish and breeding pigs. University of Minnesota Extension: St. Paul, Minnesota. https://extension.umn.edu/swine-production-management/heat-stress-swine-affects-production#sources-1697410

[CIT0028] Shinde, P., R. S. Dass, A. K. Garg, V. K. Chaturvedi, and R. Kumar. 2006. Effect of zinc supplementation from different sources on growth, nutrient digestibility, blood metabolic profile, and immune response of male Guinea pigs. Biol. Trace Elem. Res. 112:247–262. doi:10.1385/bter:112:3:247 17057264

[CIT0029] Spencer, J. D., A. M. Gaines, E. P. Berg, and G. L. Allee. 2005. Diet modifications to improve finishing pig growth performance and pork quality attributes during periods of heat stress. J. Anim. Sci. 83:243–254. doi:10.2527/2005.831243x 15583065

[CIT0030] St-Pierre, N. R., B. Cobanov, and G. Schnitkey. 2003. Economic losses from heat stress by US livestock industries. J. Dairy Sci. 86:E52–E77. doi:10.3168/jds.s0022-0302(03)74040-5

[CIT0031] Verstegen, M. W. A., E. W. Brascamp, and W. Van der Hel. 1978. Growing and fattening of pigs in relation to temperature of housing and feeding level. Can. J. Anim. Sci. 58:1–13. doi:10.4141/cjas78-001

[CIT0032] Wang, Y., J. Tang, W. Ma, J. Feng, and J. Feng. 2010. Dietary zinc glycine chelate on growth performance, tissue mineral concentrations, and serum enzyme activity in weanling piglets. Biol. Trace Elem. Res. 133:325–334. doi:10.1007/s12011-009-8437-3. 19557314

[CIT0033] Yin, J., X. Li, D. Li, T. Yue, Q. Fang, J. Ni, X. Zhou, and G. Wu. 2009. Dietary supplementation with zinc oxide stimulates ghrelin secretion from the stomach of young pigs. J. Nutr. Biochem. 20:783–790. doi:10.1016/j.jnutbio.2008.07.007 18926680

[CIT0034] Zhang, B., and Y. Guo. 2009. Supplemental zinc reduced intestinal permeability by enhancing occludin and zonula occludens protein-1 (ZO-1) expression in weaning piglets. Br. J. Nutr. 102:687–693. doi:10.1017/S0007114509289033 19267955

