# Supplementary material for: Effect of chronic cyclic heat stress and supplemented inorganic and organic zinc source levels on grow-finish pig growth performance and estimated body composition
Source: Transl Anim Sci. 2024 Apr 2;8:txae029. doi: 10.1093/tas/txae029 (PMC10999157; doi:10.1093/tas/txae029)
Supplement: txae029_suppl_Supplementary_Tables [file txae029_suppl_supplementary_tables.docx]

**Supplemental Table 1. Analyzed composition of diets^1^**

| Dry Matter, % |  |  |  |
| --- | --- | --- | --- |
| Diet^2^ | GF4 | GF5 | GF6 |
| 1 | 88.89 | 88.36 | 89.16 |
| 2 | 89.29 | 89.48 | 89.40 |
| 3 | 89.58 | 89.29 | 89.33 |
| 4 | 89.38 | 89.43 | 89.71 |
| 5 | 89.53 | 88.34 | 89.35 |
| 6 | 89.77 | 89.49 | 89.47 |
|  |  |  |  |
| Ash, % |  |  |  |
| Diet | GF4 | GF5 | GF6 |
| 1 | 4.241 | 3.669 | 3.421 |
| 2 | 4.010 | 3.6128 | 3.335 |
| 3 | 4.039 | 3.7180 | 3.304 |
| 4 | 4.053 | 3.6819 | 3.265 |
| 5 | 4.073 | 3.5469 | 3.327 |
| 6 | 3.945 | 3.6531 | 3.370 |
|  |  |  |  |
| Fat, % |  |  |  |
| Diet | GF4 | GF5 | GF6 |
| 1 | 6.680 | 6.020 | 7.066 |
| 2 | 6.189 | 6.561 | 6.332 |
| 3 | 6.735 | 6.773 | 6.270 |
| 4 | 6.100 | 6.356 | 6.212 |
| 5 | 6.209 | 6.274 | 6.643 |
| 6 | 6.888 | 6.065 | 6.860 |
|  |  |  |  |
| Energy, kcal/g |  |  |  |
| Diet | GF4 | GF5 | GF6 |
| 1 | 4.032 | 4.001 | 4.030 |
| 2 | 4.027 | 4.016 | 4.043 |
| 3 | 4.058 | 4.001 | 4.032 |
| 4 | 4.040 | 4.012 | 4.039 |
| 5 | 4.058 | 3.997 | 3.999 |
| 6 | 4.034 | 4.026 | 4.041 |
|  |  |  |  |
| Crude Protein, % |  |  |  |
| Diet | GF4 | GF5 | GF6 |
| 1 | 15.42 | 13.35 | 13.74 |
| 2 | 15.33 | 13.34 | 13.82 |
| 3 | 15.48 | 13.72 | 13.46 |
| 4 | 15.52 | 14.45 | 13.47 |
| 5 | 15.43 | 14.28 | 13.41 |
| 6 | 15.58 | 14.09 | 13.18 |
|  |  |  |  |
| Phosphorus, % |  |  |  |
| Diet | GF4 | GF5 | GF6 |
| 1 | 0.509 | 0.327 | 0.497 |
| 2 | 0.560 | 0.577 | 0.476 |
| 3 | 0.479 | 0.450 | 0.466 |
| 4 | 0.613 | 0.550 | 0.452 |
| 5 | 0.509 | 0.490 | 0.469 |
| 6 | 0.521 | 0.511 | 0.457 |
|  |  |  |  |
| Zinc mg/kg |  |  |  |
| Diet | GF4 | GF5 | GF6 |
| 1 | 114.2 | 101.2 | 108.3 |
| 2 | 177.1 | 178.5 | 191.3 |
| 3 | 92.41 | 84.76 | 98.80 |
| 4 | 125.7 | 120.0 | 163.0 |
| 5 | 144.5 | 164.0 | 181.2 |
| 6 | 178.2 | 187.13 | 206.4 |

^1^ Analyzed at Purdue University

^2^ Dietary available zinc supplementation: 1) 50 mg/kg ZnO; 2) 130 mg/kg ZnO: 3)

50 mg/kg of organic Zn (Availa^®^Zn); 4) 50 mg/kg ZnO + 40 mg/kg organic Zn);

5) 50 mg/kg ZnO + 60 mg/kg organic Zn; and 6) 50 mg/kg ZnO + 80 mg/kg organic Zn.

**Supplemental Table 2. Analyzed amino acid and proximate composition of diets for GF4^1^**

| Amino Acid, W/W%^2^ | Diet 1^3^ | Diet 2 | Diet 3 | Diet 4 | Diet 5 | Diet 6 |
| --- | --- | --- | --- | --- | --- | --- |
| Taurine | 0.20 | 0.19 | 0.20 | 0.19 | 0.20 | 0.21 |
| Hydroxyproline | 0.00 | 0.06 | 0.00 | 0.05 | 0.00 | 0.00 |
| Aspartic Acid | 1.27 | 1.31 | 1.13 | 1.21 | 1.30 | 1.13 |
| Threonine | 0.57 | 0.59 | 0.52 | 0.64 | 0.56 | 0.54 |
| Serine | 0.60 | 0.61 | 0.55 | 0.58 | 0.61 | 0.56 |
| Glutamic Acid | 2.51 | 2.54 | 2.28 | 2.40 | 2.57 | 2.28 |
| Proline | 1.23 | 1.23 | 1.19 | 1.20 | 1.25 | 1.13 |
| Glycine | 0.68 | 0.68 | 0.64 | 0.67 | 0.69 | 0.63 |
| Alanine | 0.91 | 0.9 | 0.86 | 0.88 | 0.93 | 0.85 |
| Cysteine | 0.28 | 0.27 | 0.25 | 0.23 | 0.28 | 0.24 |
| Valine | 0.73 | 0.74 | 0.67 | 0.71 | 0.74 | 0.67 |
| Methionine | 0.32 | 0.29 | 0.29 | 0.25 | 0.31 | 0.28 |
| Isoleucine | 0.63 | 0.65 | 0.56 | 0.61 | 0.64 | 0.56 |
| Leucine | 1.40 | 1.39 | 1.29 | 1.36 | 1.44 | 1.31 |
| Tyrosine | 0.51 | 0.53 | 0.46 | 0.49 | 0.52 | 0.48 |
| Phenylalanine | 0.77 | 0.81 | 0.69 | 0.74 | 0.81 | 0.72 |
| Hydroxylysine | 0.06 | 0.08 | 0.05 | 0.05 | 0.08 | 0.08 |
| Ornithine | 0.02 | 0.04 | 0.02 | 0.01 | 0.03 | 0.03 |
| Lysine | 0.97 | 1.16 | 0.91 | 0.98 | 0.97 | 0.92 |
| Histidine | 0.42 | 0.46 | 0.38 | 0.4 | 0.45 | 0.41 |
| Arginine | 0.84 | 0.90 | 0.76 | 0.82 | 0.85 | 0.76 |
| Tryptophan | 0.19 | 0.20 | 0.17 | 0.17 | 0.18 | 0.19 |
|  |  |  |  |  |  |  |
| Total | 15.1 | 15.6 | 13.9 | 14.6 | 15.4 | 14.0 |
|  |  |  |  |  |  |  |
| Nitrogen | 2.34 | 2.28 | 2.19 | 2.34 | 2.34 | 2.25 |
| Dry Matter | 87.4 | 87.6 | 88.1 | 87.5 | 88.0 | 87.5 |
| Crude Fat | 6.35 | 6.23 | 6.08 | 5.61 | 6.30 | 5.65 |
| Crude Fiber | 2.25 | 2.39 | 2.22 | 2.23 | 2.32 | 2.31 |
| Calcium | 0.52 | 0.53 | 0.61 | 0.59 | 0.56 | 0.66 |
| Phosphorus | 0.44 | 0.43 | 0.43 | 0.45 | 0.44 | 0.42 |
| Zinc mg/kg | 92.8 | 172 | 42.6 | 109 | 137 | 138 |

^1^ Analyzed at the University of Missouri

^2^ W/W%= grams per 100 grams of sample

^3^ Dietary available zinc supplementation: 1) 50 mg/kg ZnO; 2) 130 mg/kg ZnO: 3) 50 mg/kg of organic Zn (Availa^®^Zn); 4) 50 mg/kg ZnO + 40 mg/kg organic Zn); 5) 50 mg/kg ZnO + 60 mg/kg organic Zn; and 6) 50 mg/kg ZnO + 80 mg/kg organic Zn.

**Supplemental Table 3. Analyzed amino acid and proximate composition of diets for GF5^1^**

| Amino Acid, W/W%^2^ | Diet 1^3^ | Diet 2 | Diet 3 | Diet 4 | Diet 5 | Diet 6 |
| --- | --- | --- | --- | --- | --- | --- |
| Taurine | 0.20 | 0.20 | 0.20 | 0.19 | 0.20 | 0.19 |
| Hydroxyproline | 0.00 | 0.05 | 0.02 | 0.00 | 0.03 | 0.04 |
| Aspartic Acid | 1.04 | 1.05 | 0.90 | 0.99 | 0.99 | 1.25 |
| Threonine | 0.49 | 0.53 | 0.44 | 0.44 | 0.46 | 0.54 |
| Serine | 0.52 | 0.52 | 0.46 | 0.48 | 0.49 | 0.58 |
| Glutamic Acid | 2.08 | 2.07 | 1.91 | 1.97 | 1.98 | 2.32 |
| Proline | 1.09 | 1.05 | 1.00 | 1.01 | 1.02 | 1.15 |
| Glycine | 0.57 | 0.61 | 0.52 | 0.57 | 0.59 | 0.66 |
| Alanine | 0.79 | 0.83 | 0.76 | 0.78 | 0.79 | 0.89 |
| Cysteine | 0.21 | 0.25 | 0.22 | 0.21 | 0.22 | 0.28 |
| Valine | 0.61 | 0.63 | 0.56 | 0.61 | 0.61 | 0.70 |
| Methionine | 0.24 | 0.28 | 0.24 | 0.21 | 0.21 | 0.30 |
| Isoleucine | 0.50 | 0.51 | 0.45 | 0.49 | 0.48 | 0.55 |
| Leucine | 1.20 | 1.21 | 1.18 | 1.18 | 1.18 | 1.33 |
| Tyrosine | 0.45 | 0.40 | 0.41 | 0.43 | 0.43 | 0.44 |
| Phenylalanine | 0.66 | 0.63 | 0.6 | 0.66 | 0.62 | 0.65 |
| Hydroxylysine | 0.08 | 0.03 | 0.04 | 0.07 | 0.04 | 0.02 |
| Ornithine | 0.03 | 0.01 | 0.02 | 0.03 | 1.00 | 0.01 |
| Lysine | 0.85 | 0.88 | 0.81 | 0.83 | 0.83 | 0.89 |
| Histidine | 0.39 | 0.38 | 0.36 | 0.41 | 0.37 | 0.44 |
| Arginine | 0.71 | 0.68 | 0.6 | 0.71 | 0.69 | 0.81 |
| Tryptophan | 0.15 | 0.17 | 0.15 | 0.15 | 0.16 | 0.19 |
|  |  |  |  |  |  |  |
| Total | 12.9 | 13.0 | 11.9 | 12.4 | 12.4 | 14.2 |
|  |  |  |  |  |  |  |
| Nitrogen | 2.06 | 2.20 | 2.13 | 2.13 | 2.06 | 2.04 |
| Dry Matter | 87.2 | 87.3 | 87.2 | 87.0 | 87.2 | 87.2 |
| Crude Fat | 9.75 | 9.96 | 6.70 | 6.83 | 6.30 | 6.50 |
| Crude Fiber | 2.28 | 2.36 | 2.32 | 2.16 | 2.27 | 2.43 |
| Calcium | 0.58 | 0.62 | 0.57 | 0.32 | 0.56 | 0.49 |
| Phosphorus | 0.37 | 397 | 0.40 | 0.37 | 0.41 | 0.44 |
| Zinc mg/kg | 106 | 193 | 62.8 | 84.8 | 136 | 135 |

^1^ Analyzed at the University of Missouri

^2^ W/W%= grams per 100 grams of sample

^3^Dietary available zinc supplementation: 1) 50 mg/kg ZnO; 2) 130 mg/kg ZnO: 3) 50 mg/kg of organic Zn (Availa^®^Zn); 4) 50 mg/kg ZnO + 40 mg/kg organic Zn); 5) 50 mg/kg ZnO + 60 mg/kg organic Zn; and 6) 50 mg/kg ZnO + 80 mg/kg organic Zn.

**Supplemental Table 4. Analyzed amino acid and proximate composition of diets for GF6^1^**

| Amino Acid, W/W%^2^ | Diet 1^3^ | Diet 2 | Diet 3 | Diet 4 | Diet 5 | Diet 6 |
| --- | --- | --- | --- | --- | --- | --- |
| Taurine | 0.20 | 0.20 | 0.20 | 0.21 | 0.20 | 0.20 |
| Hydroxyproline | 0.06 | 0.06 | 0.00 | 0.10 | 0.00 | 0.05 |
| Aspartic Acid | 0.97 | 0.91 | 1.02 | 0.93 | 0.81 | 0.98 |
| Threonine | 0.45 | 0.50 | 0.47 | 0.44 | 0.43 | 0.48 |
| Serine | 0.49 | 0.47 | 0.50 | 0.48 | 0.45 | 0.52 |
| Glutamic Acid | 1.95 | 1.87 | 1.99 | 1.94 | 1.83 | 2.09 |
| Proline | 1.08 | 0.99 | 1.04 | 1.05 | 1.00 | 1.10 |
| Glycine | 0.55 | 0.55 | 0.56 | 0.55 | 0.53 | 0.59 |
| Alanine | 0.82 | 0.76 | 0.80 | 0.79 | 0.77 | 0.85 |
| Cysteine | 0.24 | 0.22 | 0.26 | 0.21 | 0.20 | 0.23 |
| Valine | 0.59 | 0.57 | 0.58 | 0.57 | 0.54 | 0.61 |
| Methionine | 0.31 | 0.21 | 0.25 | 0.20 | 0.20 | 0.23 |
| Isoleucine | 0.47 | 0.45 | 0.46 | 0.46 | 0.42 | 0.48 |
| Leucine | 1.25 | 1.16 | 1.20 | 1.19 | 1.14 | 1.26 |
| Tyrosine | 0.44 | 0.41 | 0.40 | 0.44 | 0.40 | 0.42 |
| Phenylalanine | 0.66 | 0.61 | 0.56 | 0.64 | 0.56 | 0.59 |
| Hydroxylysine | 0.08 | 0.05 | 0.02 | 0.08 | 0.04 | 0.02 |
| Ornithine | 0.03 | 0.02 | 0.01 | 0.03 | 0.01 | 0.01 |
| Lysine | 0.76 | 0.87 | 0.82 | 0.88 | 0.71 | 0.83 |
| Histidine | 0.40 | 0.37 | 0.38 | 0.39 | 0.35 | 0.39 |
| Arginine | 0.64 | 0.62 | 0.63 | 0.65 | 0.60 | 0.69 |
| Tryptophan | 0.13 | 0.15 | 0.16 | 0.15 | 0.16 | 0.14 |
|  |  |  |  |  |  |  |
| Total | 12.6 | 12.0 | 12.3 | 12.4 | 11.4 | 12.8 |
|  |  |  |  |  |  |  |
| Nitrogen | 1.87 | 2.03 | 1.93 | 2.07 | 2.04 | 1.91 |
| Dry Matter | 87.6 | 87.5 | 87.6 | 87.6 | 87.4 | 87.6 |
| Crude Fat | 7.02 | 5.99 | 6.49 | 5.94 | 6.55 | 8.19 |
| Crude Fiber | 2.30 | 2.20 | 2.24 | 2.18 | 2.25 | 2.27 |
| Calcium | 0.41 | 0.57 | 0.58 | 0.59 | 0.50 | 0.50 |
| Phosphorus | 0.37 | 0.35 | 0.37 | 0.35 | 0.37 | 0.39 |
| Zinc mg/kg | 108 | 196 | 115 | 120 | 210 | 314 |

^1^ Analyzed at the University of Missouri

^2^ W/W%= grams per 100 grams of sample

^3^ Dietary available zinc supplementation: 1) 50 mg/kg ZnO; 2) 130 mg/kg ZnO: 3) 50 mg/kg of organic Zn (Availa^®^Zn); 4) 50 mg/kg ZnO + 40 mg/kg organic Zn); 5) 50 mg/kg ZnO + 60 mg/kg organic Zn; and 6) 50 mg/kg ZnO + 80 mg/kg organic Zn.

**Supplemental Table 5. Main effect of dietary zinc concentration and source on grow-finish pig growth performance**

| Diet | 1 | 2 | 3 | 4 | 5 | 6 |  |
| --- | --- | --- | --- | --- | --- | --- | --- |
| ZnO/Organic Zn, mg/kg available Zn | 50/0 | 130/0 | 0/50 | 50/40 | 50/60 | 50/80 | SE |
| Initial BW, kg | 72.24 | 72.15 | 72.01 | 72.21 | 72.34 | 72.09 | 1.813 |
| Day 0-18 |  |  |  |  |  |  |  |
| ADG, kg/d | 0.853 | 0.949 | 0.887 | 0.893 | 0.897 | 0.906 | 0.043 |
| ADFI, kg/d | 2.688 | 2.760 | 2.660 | 2.682 | 2.758 | 2.722 | 0.093 |
| G:F | 0.315 | 0.340 | 0.331 | 0.331 | 0.324 | 0.329 | 0.008 |
| d 18 BW, kg | 87.60 | 88.73 | 87.97 | 88.28 | 88.50 | 88.54 | 2.267 |
| Day 18-21 |  |  |  |  |  |  |  |
| ADG, kg/d | 0.980 | 0.943 | 0.996 | 0.955 | 0.966 | 0.920 | 0.066 |
| ADFI, kg/d | 2.766 | 2.851 | 2.783 | 2.779 | 2.775 | 2.711 | 0.077 |
| G:F | 0.353 | 0.328 | 0.361 | 0.327 | 0.363 | 0.352 | 0.019 |
| d 21 BW, kg | 90.71 | 91.35 | 91.11 | 90.98 | 91.50 | 91.32 | 2.315 |
| Day 21-24 |  |  |  |  |  |  |  |
| ADG, kg/d | 0.721 | 0.894 | 0.785 | 0.803 | 0.852 | 0.882 | 0.064 |
| ADFI, kg/d | 2.834 | 2.955 | 2.872 | 2.995 | 3.015 | 2.918 | 0.063 |
| G:F | 0.256 | 0.305 | 0.281 | 0.289 | 0.281 | 0.309 | 0.022 |
| d 24 BW, kg | 92.88 | 94.23 | 93.32 | 93.39 | 94.04 | 93.89 | 2.304 |
| Day 24-42 |  |  |  |  |  |  |  |
| ADG, kg/d | 0.876 | 0.887 | 0.874 | 0.876 | 0.914 | 0.901 | 0.028 |
| ADFI, kg/d | 2.938 | 3.009 | 2.993 | 3.027 | 2.976 | 3.034 | 0.087 |
| G:F | 0.299 | 0.295 | 0.291 | 0.290 | 0.303 | 0.297 | 0.007 |
| d 42 BW, kg | 108.65 | 110.19 | 109.02 | 109.19 | 109.83 | 109.57 | 2.628 |
| Day 42-45 |  |  |  |  |  |  |  |
| ADG, kg/d | 0.901 | 0.895 | 0.774 | 0.839 | 0.736 | 0.806 | 0.054 |
| ADFI, kg/d | 2.850 | 2.922 | 2.785 | 2.984 | 2.907 | 2.987 | 0.113 |
| G:F | 0.310 | 0.307 | 0.302 | 0.304 | 0.258 | 0.307 | 0.021 |
| d 45 BW, kg | 111.22 | 112.87 | 111.49 | 111.85 | 112.03 | 112.17 | 2.576 |
| Day 45-63 |  |  |  |  |  |  |  |
| ADG, kg/d | 0.821 | 0.817 | 0.790 | 0.789 | 0.829 | 0.813 | 0.027 |
| ADFI, kg/d | 2.918 | 2.929 | 2.912 | 2.913 | 3.044 | 2.988 | 0.067 |
| G:F | 0.282 | 0.279 | 0.271 | 0.270 | 0.272 | 0.271 | 0.005 |
| d 63 BW, kg | 125.70 | 127.27 | 125.45 | 125.75 | 126.67 | 126.51 | 2.765 |
| Diet Phases |  |  |  |  |  |  |  |
| Day 0-21 (GF4) |  |  |  |  |  |  |  |
| ADG, kg/d | 0.880 | 0.914 | 0.910 | 0.894 | 0.907 | 0.909 | 0.035 |
| ADFI, kg/d | 2.693 | 2.784 | 2.678 | 2.707 | 2.760 | 2.720 | 0.087 |
| G:F | 0.326 | 0.332 | 0.339 | 0.330 | 0.328 | 0.331 | 0.006 |
| Day 21-42 (GF5) |  |  |  |  |  |  |  |
| ADG, kg/d | 0.854 | 0.897 | 0.854 | 0.867 | 0.903 | 0.899 | 0.027 |
| ADFI, kg/d | 2.923 | 3.001 | 2.976 | 3.022 | 2.982 | 3.027 | 0.077 |
| G:F | 0.292 | 0.299 | 0.286 | 0.287 | 0.300 | 0.296 | 0.007 |
| Day 42-63 (GF6) |  |  |  |  |  |  |  |
| ADG, kg/d | 0.826 | 0.828 | 0.795 | 0.803 | 0.815 | 0.821 | 0.027 |
| ADFI, kg/d | 2.908 | 2.928 | 2.893 | 2.923 | 3.024 | 2.987 | 0.070 |
| G:F | 0.284 | 0.283 | 0.274 | 0.274 | 0.269 | 0.274 | 0.005 |
| Overall |  |  |  |  |  |  |  |
| Day 0-63 |  |  |  |  |  |  |  |
| ADG, kg/d | 0.854 | 0.880 | 0.854 | 0.855 | 0.867 | 0.869 | 0.021 |
| ADFI, kg/d | 2.842 | 2.905 | 2.849 | 2.884 | 2.923 | 2.912 | 0.064 |
| G:F | 0.300 | 0.303 | 0.299 | 0.296 | 0.297 | 0.298 | 0.003 |
| Cyclic Heat Pigs Only |  |  |  |  |  |  |  |
| Day 63-66 |  |  |  |  |  |  |  |
| ADG, kg/d | 0.754 | 0.531 | 0.716 | 0.643 | 0.695 | 0.621 | 0.092 |
| ADFI, kg/d | 2.348 | 2.541 | 2.508 | 2.468 | 2.486 | 2.509 | 0.106 |
| G:F | 0.301 | 0.207 | 0.285 | 0.296 | 0.278 | 0.247 | 0.037 |
| d 63 BW, kg^1^ | 124.23 | 125.40 | 123.55 | 123.02 | 125.67 | 122.39 | 3.192 |
| d 66 BW, kg | 126.24 | 126.99 | 125.70 | 124.94 | 127.76 | 124.25 | 3.112 |
| Day 66-70 |  |  |  |  |  |  |  |
| ADG, kg/d | 0.717 | 0.723 | 0.769 | 0.951 | 0.723 | 0.796 | 0.067 |
| ADFI, kg/d | 2.883 | 2.794 | 2.784 | 2.821 | 2.803 | 2.813 | 0.125 |
| G:F | 0.247 | 0.259 | 0.280 | 0.340 | 0.253 | 0.284 | 0.021 |
| Day 63-70 |  |  |  |  |  |  |  |
| ADG, kg/d | 0.696 | 0.645 | 0.752 | 0.825 | 0.703 | 0.751 | 0.048 |
| ADFI, kg/d | 2.604 | 2.674 | 2.655 | 2.674 | 2.665 | 2.727 | 0.098 |
| G:F | 0.270 | 0.242 | 0.284 | 0.311 | 0.263 | 0.266 | 0.019 |
| d 70 BW, kg | 129.38 | 130.18 | 129.09 | 129.14 | 130.86 | 127.43 | 3.090 |
| Day 0-70 |  |  |  |  |  |  |  |
| ADG, kg/d | 0.820 | 0.833 | 0.814 | 0.806 | 0.836 | 0.793 | 0.024 |
| ADFI, kg/d | 2.719 | 2.786 | 2.679 | 2.713 | 2.792 | 2.689 | 0.067 |
| G:F | 0.301 | 0.299 | 0.303 | 0.297 | 0.299 | 0.293 | 0.005 |
| ^1^  Day 63 pen mean weight after removal of 1 pig/pen for carcass data at the Purdue University meats lab. | | | | | | | |

**Supplemental Table 6. Contrasts ran with Proc MIXED for growth performance criteria**

|  | Day 0-18 | | | | | Day 18-21 | | | |
| --- | --- | --- | --- | --- | --- | --- | --- | --- | --- |
| Contrast | Initial BW, kg | ADG, kg/d | ADFI, kg/d | G:F | d 18 BW, kg | ADG, kg/d | ADFI, kg/d | G:F | d 21 BW, kg |
| Temp^1^ | 0.4693 | 0.9884 | 0.6499 | 0.4059 | 0.7163 | 0.5823 | 0.0002 | 0.1137 | 0.8340 |
| Zn Source^2^ | 0.9561 | 0.8630 | 0.7577 | 0.8658 | 0.8503 | 0.9702 | 0.4525 | 0.5415 | 0.8070 |
| Zn Level^3^ Quadratic | 0.8321 | 0.7818 | 0.9259 | 0.9018 | 0.9338 | 0.9516 | 0.9855 | 0.6621 | 0.9939 |
| Zn Level Linear | 0.9260 | 0.1483 | 0.2387 | 0.3300 | 0.3719 | 0.4260 | 0.9448 | 0.6914 | 0.5654 |
| Source x Temp | 0.8138 | 0.3249 | 0.3674 | 0.5442 | 0.8732 | 0.6760 | 0.2900 | 0.5821 | 0.9479 |
| Level Linear x Temp | 0.9260 | 0.1483 | 0.2387 | 0.3300 | 0.3719 | 0.4260 | 0.9448 | 0.6914 | 0.5654 |
| Level Quad x Temp | 0.6903 | 0.8785 | 0.6866 | 0.6650 | 0.7667 | 0.7742 | 0.3560 | 0.5763 | 0.9351 |
| Source x Level Quad x Temp | 0.6326 | 0.4925 | 0.2122 | 0.7396 | 0.6300 | 0.3540 | 0.5614 | 0.9515 | 0.9383 |
| Source x Level Lin x Temp | 0.5818 | 0.7838 | 0.8077 | 0.6820 | 0.6043 | 0.5498 | 0.9313 | 0.9294 | 0.3868 |
| 50 InOrg vs. 50 Org | 0.8138 | 0.5148 | 0.7786 | 0.2032 | 0.7860 | 0.8509 | 0.8594 | 0.7774 | 0.7607 |
| 130 InOrg vs. 130 Org | 0.9486 | 0.4111 | 0.6966 | 0.3999 | 0.8920 | 0.7952 | 0.1683 | 0.3859 | 0.9841 |

^1^Temp = Cyclic Heat (HS) vs Thermoneutral (TN).

^2^Source = Inorganic vs Organic; diets 1, 2 vs 3, 4, 5, 6.

^3^Contrast “Level” (Level) = Total level available Zn regardless of source of either 50 (2 diets), 90, 110, 130 (2 diets); diets 1, 3 vs 4 vs 5 vs 2, 6

|  | Day 21-24 | | | | Day 24-42 | | | |
| --- | --- | --- | --- | --- | --- | --- | --- | --- |
| Contrast | ADG, kg/d | ADFI, kg/d | G:F | d 24 BW, kg | ADG, kg /d | ADFI, kg /d | G:F | d 42 BW, kg |
| Temp^1^ | <.0001 | <.0001 | 0.0306 | 0.2348 | 0.0050 | <.0001 | 0.0127 | 0.0227 |
| Zn Source^2^ | 0.6781 | 0.3178 | 0.6284 | 0.8850 | 0.6841 | 0.5208 | 0.7893 | 0.9864 |
| Zn Level^3^ Quadratic | 0.8210 | 0.0834 | 0.8067 | 0.9512 | 0.9168 | 0.8274 | 0.9045 | 0.9645 |
| Zn Level Linear | 0.0332 | 0.0933 | 0.1296 | 0.2306 | 0.2930 | 0.4950 | 0.4928 | 0.2833 |
| Source x Temp | 0.4397 | 0.0107 | 0.7826 | 0.6597 | 0.8316 | 0.2273 | 0.0726 | 0.7850 |
| Level Linear x Temp | 0.0332 | 0.0933 | 0.1296 | 0.2306 | 0.2930 | 0.4950 | 0.4928 | 0.2833 |
| Level Quad x Temp | 0.9261 | 0.5575 | 0.5769 | 0.9202 | 0.7640 | 0.5689 | 0.2196 | 0.5703 |
| Source x Level Quad x Temp | 0.0073 | <.0001 | 0.4530 | 0.7363 | 0.0964 | 0.0018 | 0.4765 | 0.4499 |
| Source x Level Lin x Temp | 0.0604 | 0.2221 | 0.1304 | 0.6760 | 0.4926 | 0.7503 | 0.1380 | 0.5096 |
| 50 InOrg vs. 50 Org | 0.4807 | 0.6774 | 0.4303 | 0.7186 | 0.9388 | 0.5235 | 0.3678 | 0.8063 |
| 130 InOrg vs. 130 Org | 0.9007 | 0.6771 | 0.9066 | 0.7882 | 0.7053 | 0.7764 | 0.8248 | 0.6838 |

^1^Temp = Cyclic Heat (HS) vs Thermoneutral (TN).

^2^Source = Inorganic vs Organic; diets 1, 2 vs 3, 4, 5, 6.

^3^Contrast “Level” (Level) = Total level available Zn regardless of source of either 50 (2 diets), 90, 110, 130 (2 diets); diets 1, 3 vs 4 vs 5 vs 2, 6

|  | Day 42-45 | | | | Day 45-63 | | | |
| --- | --- | --- | --- | --- | --- | --- | --- | --- |
| Contrast | ADG, kg /d | ADFI, kg /d | G:F | d 45 BW, kg | ADG, kg /d | ADFI, kg /d | G:F | d 63 BW, kg |
| Temp^1^ | <.0001 | <.0001 | 0.0477 | 0.0043 | <.0001 | <.0001 | 0.0049 | 0.0002 |
| Zn Source^2^ | 0.0218 | 0.7641 | 0.2795 | 0.8678 | 0.5706 | 0.4879 | 0.0434 | 0.7469 |
| Zn Level^3^ Quadratic | 0.4091 | 0.5508 | 0.2344 | 0.9025 | 0.7149 | 0.7839 | 0.3411 | 0.8222 |
| Zn Level Linear | 0.6447 | 0.2797 | 0.3639 | 0.3175 | 0.5153 | 0.2382 | 0.7406 | 0.3093 |
| Source x Temp | 0.7805 | 0.5859 | 0.2538 | 0.7954 | 0.1611 | 0.3848 | 0.1009 | 0.4831 |
| Level Linear x Temp | 0.6447 | 0.2797 | 0.3639 | 0.3175 | 0.5153 | 0.2382 | 0.7406 | 0.3093 |
| Level Quad x Temp | 0.7362 | 0.4116 | 0.5392 | 0.6896 | 0.8047 | 0.9167 | 0.5819 | 0.8149 |
| Source x Level Quad x Temp | 0.0350 | 0.0106 | 0.6450 | 0.2384 | 0.1206 | <.0001 | 0.0164 | 0.1428 |
| Source x Level Lin x Temp | 0.0155 | 0.5364 | 0.0502 | 0.7739 | 0.5882 | 0.3115 | 0.7076 | 0.9713 |
| 50 InOrg vs. 50 Org | 0.1044 | 0.6835 | 0.7500 | 0.8646 | 0.4342 | 0.9445 | 0.1367 | 0.9003 |
| 130 InOrg vs. 130 Org | 0.2481 | 0.6882 | 0.9929 | 0.6562 | 0.9282 | 0.5380 | 0.2884 | 0.6981 |

^1^Temp = Cyclic Heat (HS) vs Thermoneutral (TN).

^2^Source = Inorganic vs Organic; diets 1, 2 vs 3, 4, 5, 6.

^3^Contrast “Level” (Level) = Total level available Zn regardless of source of either 50 (2 diets), 90, 110, 130 (2 diets); diets 1, 3 vs 4 vs 5 vs 2, 6

| Diet Phases | Day 0-21 (GF4) | | | Day 21-42 (GF5) | | | Day 42-63 (GF6) | | |
| --- | --- | --- | --- | --- | --- | --- | --- | --- | --- |
| Contrast | ADG, kg/d | ADFI, kg /d | G:F | ADG, kg /d | ADFI, kg /d | G:F | ADG, kg /d | ADFI, kg /d | G:F |
| Temp^1^ | 0.6248 | 0.7158 | 0.7138 | <.0001 | <.0001 | 0.4757 | <.0001 | <.0001 | 0.1269 |
| Zn Source^2^ | 0.7396 | 0.6838 | 0.5472 | 0.8366 | 0.4299 | 0.5428 | 0.4468 | 0.5250 | 0.0200 |
| Zn Level^3^ Quadratic | 0.8165 | 0.9961 | 0.6084 | 0.9714 | 0.6862 | 0.6538 | 0.6557 | 0.7143 | 0.1753 |
| Zn Level Linear | 0.5494 | 0.2240 | 0.6708 | 0.0667 | 0.3390 | 0.0994 | 0.6109 | 0.2251 | 0.5605 |
| Source x Temp | 0.6986 | 0.3558 | 0.9460 | 0.7246 | 0.1210 | 0.3148 | 0.2372 | 0.5605 | 0.1087 |
| Level Linear x Temp | 0.5494 | 0.2240 | 0.6708 | 0.0667 | 0.3390 | 0.0994 | 0.6109 | 0.2251 | 0.5605 |
| Level Quad x Temp | 0.8498 | 0.6319 | 0.3212 | 0.8353 | 0.5854 | 0.4057 | 0.6272 | 0.9176 | 0.2353 |
| Source x Level Quad x Temp | 0.7751 | 0.3917 | 0.2651 | 0.0349 | 0.0004 | 0.8147 | 0.0293 | <.0001 | 0.1903 |
| Source x Level Lin x Temp | 0.5065 | 0.7765 | 0.7103 | 0.8921 | 0.5943 | 0.7057 | 0.2567 | 0.3315 | 0.3933 |
| 50 InOrg vs. 50 Org | 0.4648 | 0.8593 | 0.1058 | 0.9966 | 0.5166 | 0.5168 | 0.4302 | 0.8788 | 0.1702 |
| 130 InOrg vs. 130 Org | 0.9190 | 0.4798 | 0.9048 | 0.9680 | 0.7495 | 0.7185 | 0.8556 | 0.5531 | 0.2417 |

^1^Temp = Cyclic Heat (HS) vs Thermoneutral (TN).

^2^Source = Inorganic vs Organic; diets 1, 2 vs 3, 4, 5, 6.

^3^Contrast “Level” (Level) = Total level available Zn regardless of source of either 50 (2 diets), 90, 110, 130 (2 diets); diets 1, 3 vs 4 vs 5 vs 2, 6

| Overall | Day 0-63 | | |
| --- | --- | --- | --- |
| Contrast | ADG, kg /d | ADFI, kg /d | G:F |
| Temp^1^ | <.0001 | <.0001 | 0.1204 |
| Zn Source^2^ | 0.7279 | 0.6517 | 0.1347 |
| Zn Level^3^ Quadratic | 0.6975 | 0.7337 | 0.1937 |
| Zn Level Linear | 0.2663 | 0.1239 | 0.9555 |
| Source x Temp | 0.3534 | 0.1750 | 0.9223 |
| Level Linear x Temp | 0.2663 | 0.1239 | 0.9555 |
| Level Quad x Temp | 0.9626 | 0.9629 | 0.9474 |
| Source x Level Quad x Temp | 0.0495 | 0.0020 | 0.5224 |
| Source x Level Lin x Temp | 0.7806 | 0.5596 | 0.7446 |
| 50 InOrg vs. 50 Org | 0.9969 | 0.9190 | 0.7866 |
| 130 InOrg vs. 130 Org | 0.6765 | 0.9118 | 0.2683 |

^1^Temp = Cyclic Heat (HS) vs Thermoneutral (TN).

^2^Source = Inorganic vs Organic; diets 1, 2 vs 3, 4, 5, 6.

^3^Contrast “Level” (Level) = Total level available Zn regardless of source of either 50 (2 diets), 90, 110, 130 (2 diets); diets 1, 3 vs 4 vs 5 vs 2, 6

**Supplemental Table 7. Contrasts on growth performance (Cyclic Heat Pigs Only)**

| Cyclic Heat Pigs Only | Day 63-66 | | | | | Day 66-70 | | |
| --- | --- | --- | --- | --- | --- | --- | --- | --- |
| Contrasts | ADG, kg /d | ADFI, kg /d | G:F | d 63 BW, kg^1^ | d 66 BW, kg | ADG, kg /d | ADFI, kg /d | G:F |
| Zn Source^1^ | 0.7539 | 0.5406 | 0.7053 | 0.3945 | 0.4872 | 0.1179 | 0.7718 | 0.0524 |
| Zn Level^2^ Quadratic | 0.8324 | 0.9170 | 0.3613 | 0.9399 | 0.8915 | 0.0373 | 0.9646 | 0.0220 |
| Zn Level Linear | 0.1409 | 0.3313 | 0.0636 | 0.5712 | 0.6899 | 0.7592 | 0.8103 | 0.7263 |
| 50 InOrg vs. 50 Org | 0.7497 | 0.2486 | 0.6159 | 0.6268 | 0.6835 | 0.5660 | 0.5567 | 0.2692 |
| 130 InOrg vs. 130 Org | 0.4731 | 0.8541 | 0.5779 | 0.2243 | 0.2792 | 0.4701 | 0.8524 | 0.4621 |

^1^ Day 63 pen mean weight after removal of 1 pig/pen for carcass data at the Purdue University meats lab.

| Cyclic Heat Pigs Only | Day 63-70 | | | | Day 0-70 | | |
| --- | --- | --- | --- | --- | --- | --- | --- |
| Contrasts | ADG, kg /d | ADFI, kg /d | G:F | d 70 BW, kg | ADG, kg /d | ADFI, kg /d | G:F |
| Zn Source^1^ | 0.0351 | 0.5953 | 0.0972 | 0.6156 | 0.5085 | 0.4307 | 0.5885 |
| Zn Level^2^ Quadratic | 0.0777 | 0.9940 | 0.0566 | 0.6026 | 0.9390 | 0.6487 | 0.8770 |
| Zn Level Linear | 0.3941 | 0.4743 | 0.1350 | 0.7871 | 0.8443 | 0.3166 | 0.2028 |
| 50 InOrg vs. 50 Org | 0.3984 | 0.6821 | 0.5786 | 0.7562 | 0.8737 | 0.5786 | 0.7410 |
| 130 InOrg vs. 130 Org | 0.1218 | 0.6968 | 0.3096 | 0.2749 | 0.2434 | 0.1785 | 0.3055 |

^1^Source = Inorganic vs Organic; diets 1, 2 vs 3, 4, 5, 6.

^2^Contrast “Level” (Level) = Total level available Zn regardless of source of either 50 (2 diets), 90, 110, 130 (2 diets); diets 1, 3 vs 4 vs 5 vs 2, 6

**Supplemental Table 8. Contrasts for live pig body composition using real-time ultrasound**

|  | Last Rib, Back Fat, mm | 10th Rib, Back Fat, mm | Loin Muscle Depth, cm | Loin Muscle Area, cm^2^ | Body Weight D63, kg | Calculated % lean |
| --- | --- | --- | --- | --- | --- | --- |
| Temp^1^ | 0.020 | 0.001 | 0.016 | 0.001 | <0.001 | 0.095 |
| Zn Source^2^ | 0.410 | 0.218 | 0.251 | 0.655 | 0.714 | 0.681 |
| Zn Level^3^ Quadratic | 0.347 | 0.350 | 0.754 | 0.575 | 0.850 | 0.486 |
| Zn Level Linear | 0.186 | 0.365 | 0.294 | 0.746 | 0.275 | 0.229 |
| Source x Temp | 0.686 | 0.663 | 0.737 | 0.696 | 0.504 | 0.601 |
| Level Linear x Temp | 0.750 | 0.507 | 0.570 | 0.398 | 0.644 | 0.657 |
| Source x Level Quad x Temp | 0.556 | 0.207 | 0.079 | 0.149 | 0.139 | 0.693 |
| Source x Level Lin x Temp | 0.966 | 0.754 | 0.495 | 0.143 | 0.999 | 0.288 |
| 50 InOrg vs. 50 Org | 0.531 | 0.303 | 0.547 | 0.820 | 0.815 | 0.755 |
| 130 InOrg vs. 130 Org | 0.862 | 0.895 | 0.274 | 0.841 | 0.699 | 0.774 |

^1^Temp = Cyclic Heat (HS) vs Thermoneutral (TN).

^2^Source = Inorganic vs Organic; diets 1, 2 vs 3, 4, 5, 6.

^3^Contrast "Level" (Level) = Total level available Zn regardless of source of either 50 (2 diets), 90, 110, 130 (2 diets); diets 1, 3 vs 4 vs 5 vs 2, 6
